# Comparative Metagenomic Analysis of Biosynthetic Diversity across Sponge Microbiomes Highlights Metabolic Novelty, Conservation, and Diversification

**DOI:** 10.1128/msystems.00357-22

**Published:** 2022-07-18

**Authors:** Catarina Loureiro, Anastasia Galani, Asimenia Gavriilidou, Maryam Chaib de Mares, John van der Oost, Marnix H. Medema, Detmer Sipkema

**Affiliations:** a Laboratory of Microbiology, Wageningen University, Wageningen, the Netherlands; b Groningen Institute for Evolutionary Life Sciences (GELIFES), University of Groningen, Groningen, the Netherlands; c Bioinformatics Group, Wageningen University, Wageningen, the Netherlands; University of California—Santa Cruz

**Keywords:** bioinformatics, marine microbiology, marine sponge, metagenomics, natural products

## Abstract

Marine sponges and their microbial symbiotic communities are rich sources of diverse natural products (NPs) that often display biological activity, yet little is known about the global distribution of NPs and the symbionts that produce them. Since the majority of sponge symbionts remain uncultured, it is a challenge to characterize their NP biosynthetic pathways, assess their prevalence within the holobiont, and measure the diversity of NP biosynthetic gene clusters (BGCs) across sponge taxa and environments. Here, we explore the microbial biosynthetic landscapes of three high-microbial-abundance (HMA) sponges from the Atlantic Ocean and the Mediterranean Sea. This data set reveals striking novelty, with <1% of the recovered gene cluster families (GCFs) showing similarity to any characterized BGC. When zooming in on the microbial communities of each sponge, we observed higher variability of specialized metabolic and taxonomic profiles between sponge species than within species. Nonetheless, we identified conservation of GCFs, with 20% of sponge GCFs being shared between at least two sponge species and a GCF core comprised of 6% of GCFs shared across all species. Within this functional core, we identified a set of widespread and diverse GCFs encoding nonribosomal peptide synthetases that are potentially involved in the production of diversified ether lipids, as well as GCFs putatively encoding the production of highly modified proteusins. The present work contributes to the small, yet growing body of data characterizing NP landscapes of marine sponge symbionts and to the cryptic biosynthetic potential contained in this environmental niche.

**IMPORTANCE** Marine sponges and their microbial symbiotic communities are a rich source of diverse natural products (NPs). However, little is known about the sponge NP global distribution landscape and the symbionts that produce them. Here, we make use of recently developed tools to perform untargeted mining and comparative analysis of sponge microbiome metagenomes of three sponge species in the first study considering replicate metagenomes of multiple sponge species. We present an overview of the biosynthetic diversity across these sponge holobionts, which displays extreme biosynthetic novelty. We report not only the conservation of biosynthetic and taxonomic diversity but also a core of conserved specialized metabolic pathways. Finally, we highlight several novel GCFs with unknown ecological function, and observe particularly high biosynthetic potential in *Acidobacteriota* and *Latescibacteria* symbionts. This study paves the way toward a better understanding of the marine sponge holobionts’ biosynthetic potential and the functional and ecological role of sponge microbiomes.

## INTRODUCTION

Marine sponges (*Porifera*) are benthic heterotrophic filter feeders that harbor diverse and abundant communities of microbial symbionts in their tissues (1, 2). These communities have aided marine sponges in their expansion across diverse ecological niches (2–4), and are often dominated by *Proteobacteria*, *Acidobacteria*, and *Chloroflexi*, as well as the sponge-specific *Poribacteria* (5–7). The complex unit of a sponge and its microbial consortium is referred to as a “holobiont” (8) and can be divided into two categories based on the abundance and diversity of microbes in the sponge tissue, with high-microbial-abundance (HMA) sponges harboring richer and more diverse communities (7, 9–11) at 10^8^ to 10^10^ microbial cells g^−1^ sponge wet weight (5, 7) and low-microbial-abundance (LMA) sponges hosting on average 10^5^ to 10^6^ microbes g^−1^ sponge wet weight (9). Although there are exceptions to this rule (12), there is commonly an enrichment of *Poribacteria*, *Chloroflexi*, and *Acidobacteria* in HMA sponges (11). In addition, there appears to be a functional microbial core that displays gene abundance differences in core metabolic functions (13, 14).

Natural products (NPs) are ubiquitous small molecules that play a key role in symbiosis as mediators of interactions within the holobiont (15, 16). In this generally mutualistic relationship, the microbes provide their host with primary nutrients and chemical compounds that prevent predation, fouling, and infection, while receiving primary metabolic nutrition and a hospitable habitat (17–22). While symbiosis often leads to the reduction and specialization of a symbiont’s genome, the sponge holobiont specialized metabolism is maintained through positive selective pressure (23, 24). This specialization can also lead to the generation of “super producers” with high numbers of biosynthetic gene clusters, such as the genus “*Candidatus* Enthotheonella,” that was first discovered in sponges (15, 25). Sponge symbionts are a particularly prolific source of diverse NPs that often display biological activity (3, 26, 27). However, the large majority of sponge symbionts remain uncultured, which has hindered characterization of NP-mediated host-symbiont interactions (28, 29) and, consequently, access to this untapped reservoir of a broad spectrum of bacterial specialized metabolites (30, 31).

The enzymes that catalyze the production of specific specialized metabolites are generally encoded in biosynthetic gene clusters (BGCs) (32). BGCs discovered in marine symbiotic systems display a high degree of diversity, with noncanonical cluster architectures that allow for the biosynthesis of highly diverse specialized metabolites (25, 30, 33, 34). Still, there are a relatively few studies examining the sponge holobiont with a focus on specialized metabolism (30, 35, 36). Metagenomics aids in recovering environmental genetic material from uncultured microbes, and reconstruction of metagenome-assembled genomes (MAGs) facilitates studying these gene clusters within their genomic and taxonomic context (37–39). Recent development of tools such as antiSMASH (40) and BiG-SCAPE (41) (the most widely used tools for prediction and comparison of BGCs), as well as MIBiG (32; a curated database for BGCs with experimentally determined products), allow for efficient leveraging of metagenomic sequencing data sets for discovery of BGCs. This approach has revealed the existence of uncharacterized microbes with diverse specialized metabolism repertoires (42, 43) and facilitated linking putative specialized metabolites to their bacterial producers (31, 33, 44).

Untargeted mining of sponge microbiome metagenomes allows for a more complete view of the holobiont’s biosynthetic diversity and conservation across sponge holobionts (15). While NPs have mostly been described as highly niche-specialized metabolites, there are emerging data pointing to the conservation of certain BGCs in a symbiotic context (15, 45). A broader view on the extent to which BGCs are shared between the symbiotic communities of different sponge species, however, is still missing. Here, we make use of these culture-independent methods to explore the combined taxonomic and biosynthetic landscape of select marine sponge bacterial symbiont communities. In this way, we establish a detailed overview of specialized metabolic diversity in these sponge holobiont, which comprises a diverse array of mostly uncharacterized gene cluster families (GCFs). The ubiquity of a part of this array supports the hypothesis of an ecologically important set of specialized metabolic pathways conserved among the bacterial symbionts of HMA sponge species. This includes a novel set of nonribosomal peptide synthetase (NRPS)-like GCFs associated with the production of molecules related to vinyl ether lipid phosphatidylethanolamine (VEPE) (46), as well as multiple GCFs spanning several ribosomally synthesized and posttranslationally modified peptide (RiPP) classes that appear unique to sponge microbiota. In addition, we identified the putative bacterial hosts of these BGCs, which constitutes an important next step toward accessing the biosynthetic potential that is still largely untapped in sponge microbiomes.

## RESULTS AND DISCUSSION

### Sponges harbor a striking number of novel biosynthetic gene clusters.

**FIG 1:**
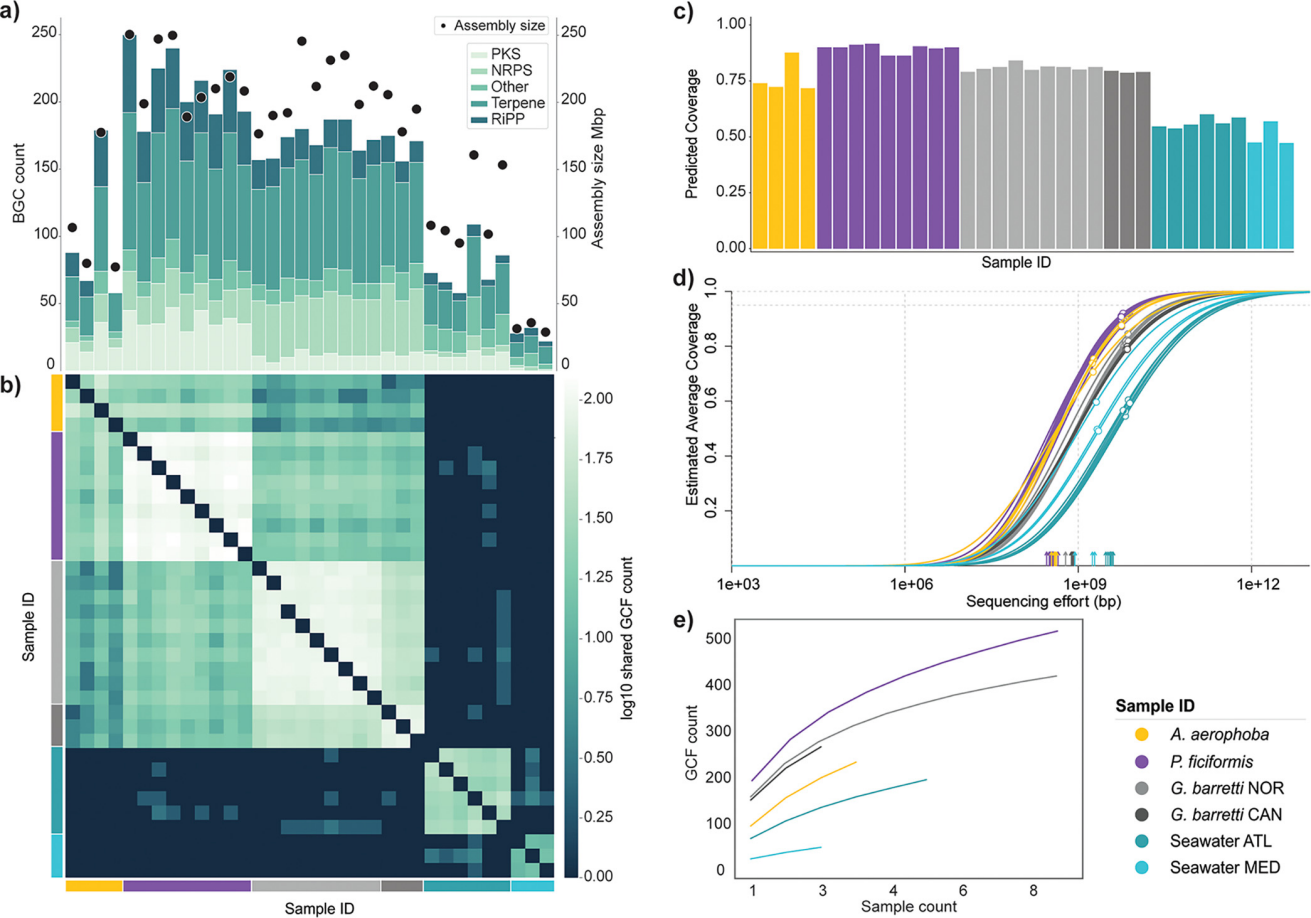
BGCs and GCFs in marine sponges. (a) BGC counts colored per major class (right *y*-axis, stacked bars) and assembly size (left *y*-axis, black dots) per sample. Assembly size includes only contigs >4,000 bp in length, used as input for antiSMASH. (b) Log_10_-normalized pairwise heatmap of shared GCF counts between samples. (c) Nonpareil sequence coverage estimates. (d) Nonpareil curves estimating the relationship between estimated coverage (*y*-axis; white dots indicate sample estimated coverage) and sequencing effort (*x*-axis; vertical arrows indicate sample sequencing effort). (e) GCF rarefaction curves.

In total, 5,082 BGCs were detected in the sponge and seawater samples, which were grouped into 1,186 GCFs plus 394 singletons. Of all the recovered GCFs, only four included experimentally characterized reference BGCs from MIBiG (47), a striking display of the potential novelty contained within marine sponges. We can observe differences between the bacterial communities of all sponge species with regard to their BGC counts, as well as sequencing depth and assembly size (Fig. 1a). The extended period between samplings and the diverse nature of the sequencing performed for each sample set warranted looking into a possible impact of the type of sequencing performed on sequence coverage and genomic content recovery. Although BGC count patterns and assembly size/sequencing effort do follow the Nonpareil estimated coverage, the latter shows less amplitude in variation (Fig. 1c and d). This indicates that BGC count patterns (Fig. 1a) observed are largely representative of the samples’ inherent sequence diversity.

Since BGC boundaries are notoriously difficult to define and BGCs are often fragmented, we have considered the GCF here to be the biosynthetic unit of study in order to minimize inflation originating from fragmented BGCs. Specialized metabolic similarity between samples can then be estimated based on the fraction of shared GCFs (Fig. 1b), with sponge samples showing high similarity even across species and habitat locations, but not to seawater samples (Fig. 1b). GCF discovery rarefaction curves (Fig. 1e) show that the biosynthetic diversity in the HMA sponge holobiont is not fully captured and indicate that a higher sequencing effort would be needed for complete coverage. Nonpareil projection curves (Fig. 1d) estimate the required sequencing effort at 10 to 100 Gbp for 90% sequence diversity recovery for HMA sponge samples. However, with estimated coverage values above 75% for all sponge samples, our data recover a large fraction of this diversity, allowing characterization of the majority of the biosynthetic landscape of these holobionts.

**FIG 2:**
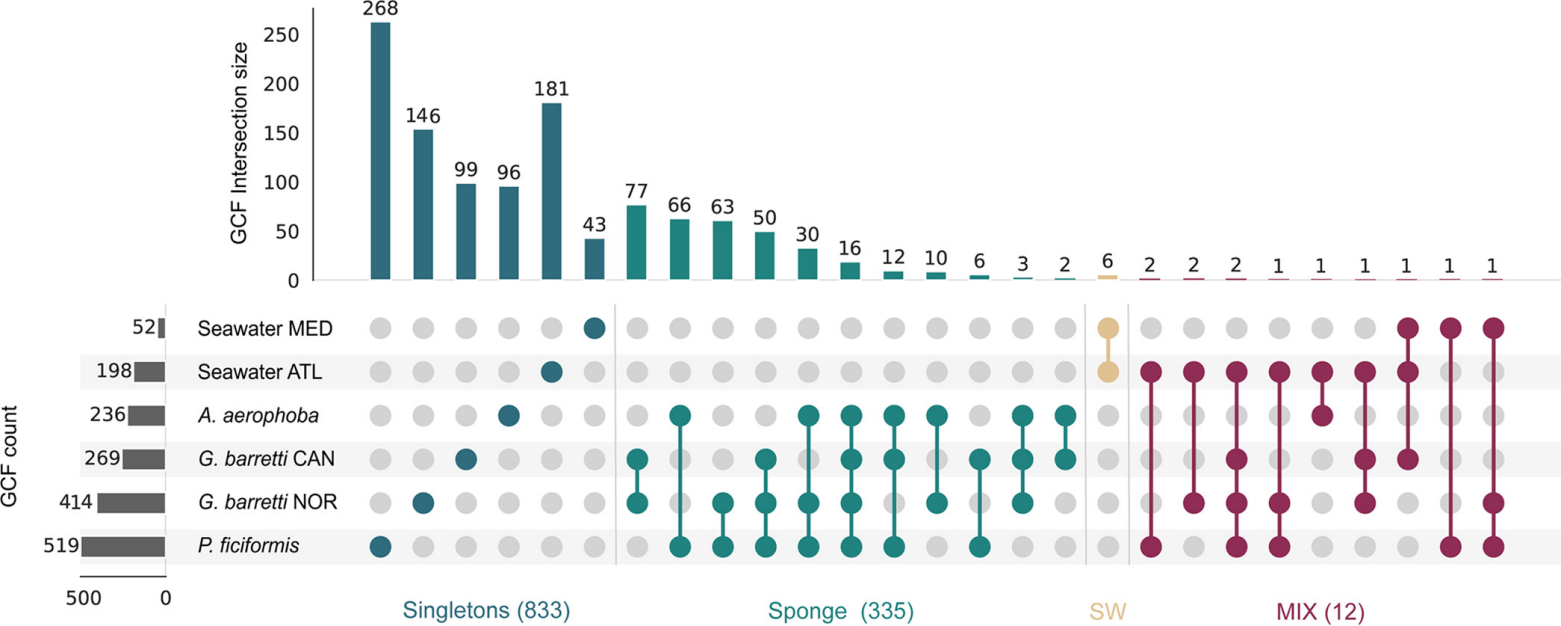
GCF intersections across sample groups. AN UpsetPlot illustrates GCF intersections between sample groups, i.e., samples grouped by sponge species and sampling geographical location. The GCF count refers to the total count for each sample group, and the intersection size refers to the GCF count of each fraction/intersection (grid dots). For each category (singletons, sponge, mix of sponge and seawater) with >1 intersection, the total GCF count is shown in parentheses.

When investigating the distribution of GCFs across the data set we see that each sponge species retains a large fraction of unique GCFs (65% of all sponge symbiont GCFs). Furthermore, there is also significant individuality between the two *Geodia barretti* sample groups, i.e., samples from two different geographical locations (Norway and Canada), with unique GCFs outnumbering shared GCFs in each of the geographical locations. Nevertheless, there is also an indication of specialized metabolite conservation across the symbiotic communities of these sponge species, with all sample groups (treating *G. barretti* from Norway and Canada as separate sample groups) sharing a core of 2% (16 GCFs) of all their encoded GCFs (Fig. 2). A total of 58 GCFs were conserved across the three sponge species (merging the two *G. barretti* groups), and we recorded that an additional 200 GCFs were shared between at least two of the three sponge species. In the sponge holobiont, functional redundancy has previously been identified for primary metabolism, with nutritionally specialized guilds that span several taxonomic affiliations (48). With regard to specialized metabolism, conservation across species has been shown with the sponge ubiquitous polyketide (SUP) cluster (49), the sponge widespread fatty acid synthase (swf) cluster (50), and the sponge derived RiPP proteusins (srp) (15). Furthermore, Mohanty et al. (51). recently reported that the presence of bromotyrosine alkaloids, signature NPs that are present across phylogenetically distant sponges, is not dependent on the sponge microbiome taxonomic architecture. Although the GCF core mentioned above is mainly composed of terpene GCFs, it also includes NRPS, RiPP, and PKS GCFs. We also recovered both SUP gene clusters (49), *swf*-like gene clusters (50) (see Fig. S1 in the supplemental material), and NRPS-like ether-lipid related GCFs (discussed below [see Fig. 5]) from all sponge species, as well as RiPP srp-like clusters from two of the three species (discussed below [see Fig. 6]).

10.1128/msystems.00357-22.1 FIG S1 SUP-like and *swf*-like GCFs. (a) SUP and *swf*-related clans in BiG-SCAPE PKSI network. (b) GCF composition of SUP and *swf*-like GCF examples. Download FIG S1, PDF file, 2.8 MB. Copyright © 2022 Loureiro et al. 2022 Loureiro et al. https://creativecommons.org/licenses/by/4.0/ This content is distributed under the terms of the Creative Commons Attribution 4.0 International license.

### Taxonomic diversity and biosynthetic gene cluster family diversity follow similar trends.

**FIG 3:**
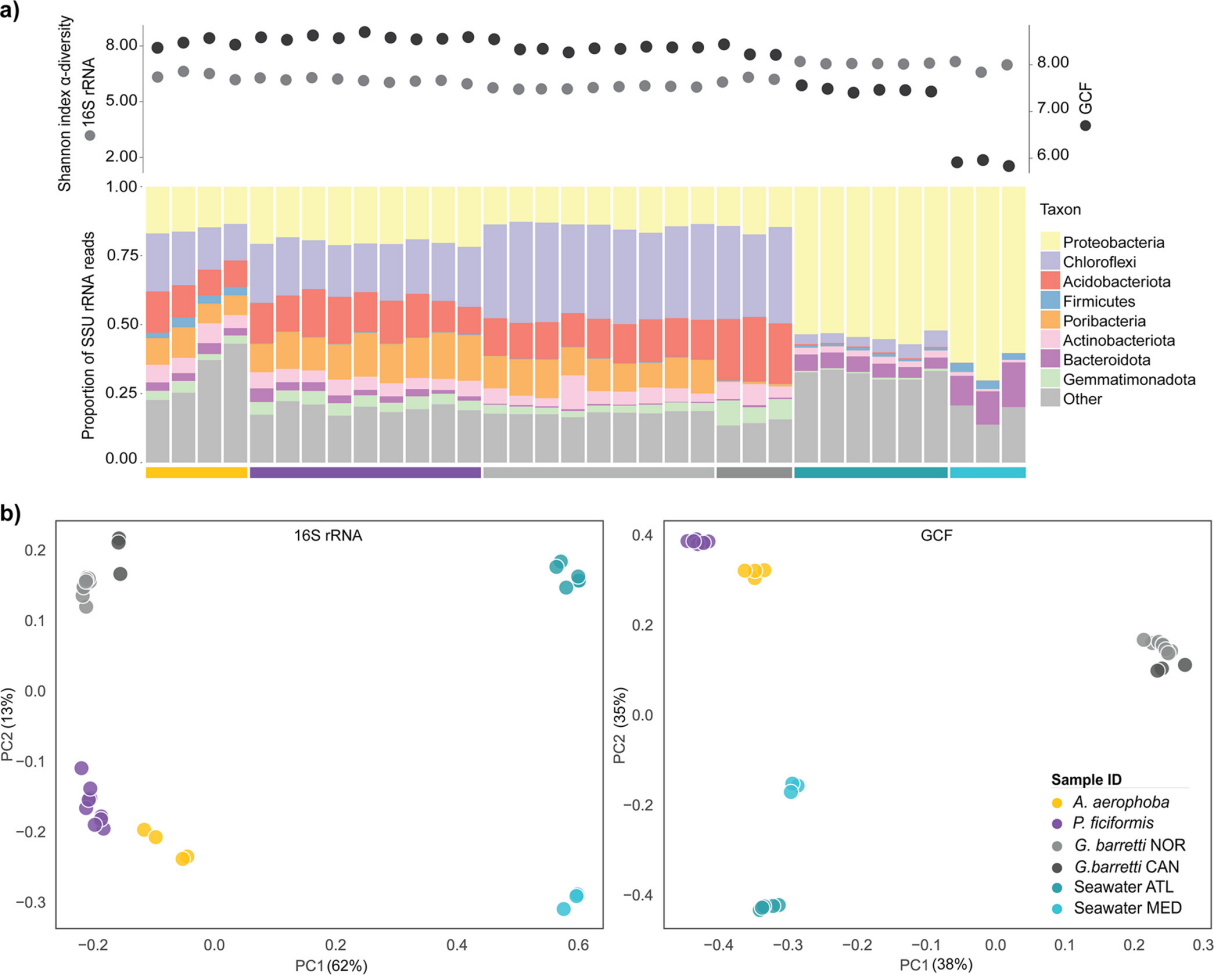
Biosynthetic potential and taxonomic diversity comparison of marine sponges. (a) Sample Shannon index alpha-diversity scores based on 16S SSU rRNA genus level NTU (left axis, light gray) and GCF (right axis, dark gray) content, and sample prokaryotic taxonomic profilea classified at the phylum level. (b) Beta-diversity scores for sponge samples based on 16S SSU rRNA genus level NTU (left) and GCF (right) content. The percent explained variance is denoted on each axis label.

As it remains unclear from which taxa in the holobiont the predicted BGCs and GCFs derive, we aimed to gain insight into the relations between functional gene content and taxonomic diversity. Observed prokaryotic community composition (Fig. 3a) follows the commonly observed profiles (52, 53), with *Proteobacteria*, *Chloroflexi*, *Acidobacteria*, and *Poribacteria* occupying the largest fractions of these sponge-associated bacterial communities.

GCF abundance-based Shannon alpha-diversity scores do not follow taxonomic 16S SSU rRNA genus level-based alpha-diversity scores across the data set (Fig. 3a), indicating that a higher diversity of bacterial genera does not automatically lead to a more diverse biosynthetic profile (Spearman’s *r* = −0.4, *P = *0.01). Furthermore, we observe that there are significant pairwise differences (Kruskal-Wallis, *P* < 0.05) between all sponge species’ taxonomy-based alpha-diversity scores, as well as GCF-based alpha-diversity scores, with *Aplysina aerophoba* showing the highest mean taxonomy-based alpha-diversity and *Petrosia ficiformis* showing the highest mean GCF-based alpha-diversity (see Table S2). Despite the significant differences in taxonomy-based alpha-diversity, GCF abundance-based alpha-diversity did not show significant differences for the *G. barretti* NOR/CAN pair (see Table S2). This is again an indication that not all bacterial genera are equally talented, since higher taxonomic diversity (*G. barretti* CAN) does not automatically lead to higher biosynthetic diversity (*G. barretti* NOR; Table S2).

10.1128/msystems.00357-22.5 TABLE S2 Alpha- and beta-diversity statistical tests. Download Table S2, PDF file, 0.2 MB. Copyright © 2022 Loureiro et al. 2022 Loureiro et al. https://creativecommons.org/licenses/by/4.0/ This content is distributed under the terms of the Creative Commons Attribution 4.0 International license.

With respect to the beta-diversity of prokaryotic community composition and GCF composition, we observed that all sample groups are different, with taxonomy and GCF content generating similar patterns (Fig. 3b). The shallow Mediterranean species, *A. aerophoba* and *P. ficiformis*, have relatively similar prokaryotic communities and GCF distributions that are different from those of the deep Atlantic sponge *G. barretti* from Norway and Canada. In addition, the prokaryotic communities and GCF distribution in seawater were different for Atlantic and Mediterranean seawater and also different than those of the three sponge species. PERMANOVA (permutational multivariate analysis of variance) testing based on 16S rRNA gene genus-level nearest taxonomic unit (NTU) and GCF content revealed that all sample groups are significantly different (*P = *0.001) in both contexts (see Table S2). These results further support the notion that, despite the presence of a shared GCF core, each sponge species has a distinct encoded specialized metabolism profile.

### *Acidobacteriota* stand out as potential superproducers.

**FIG 4:**
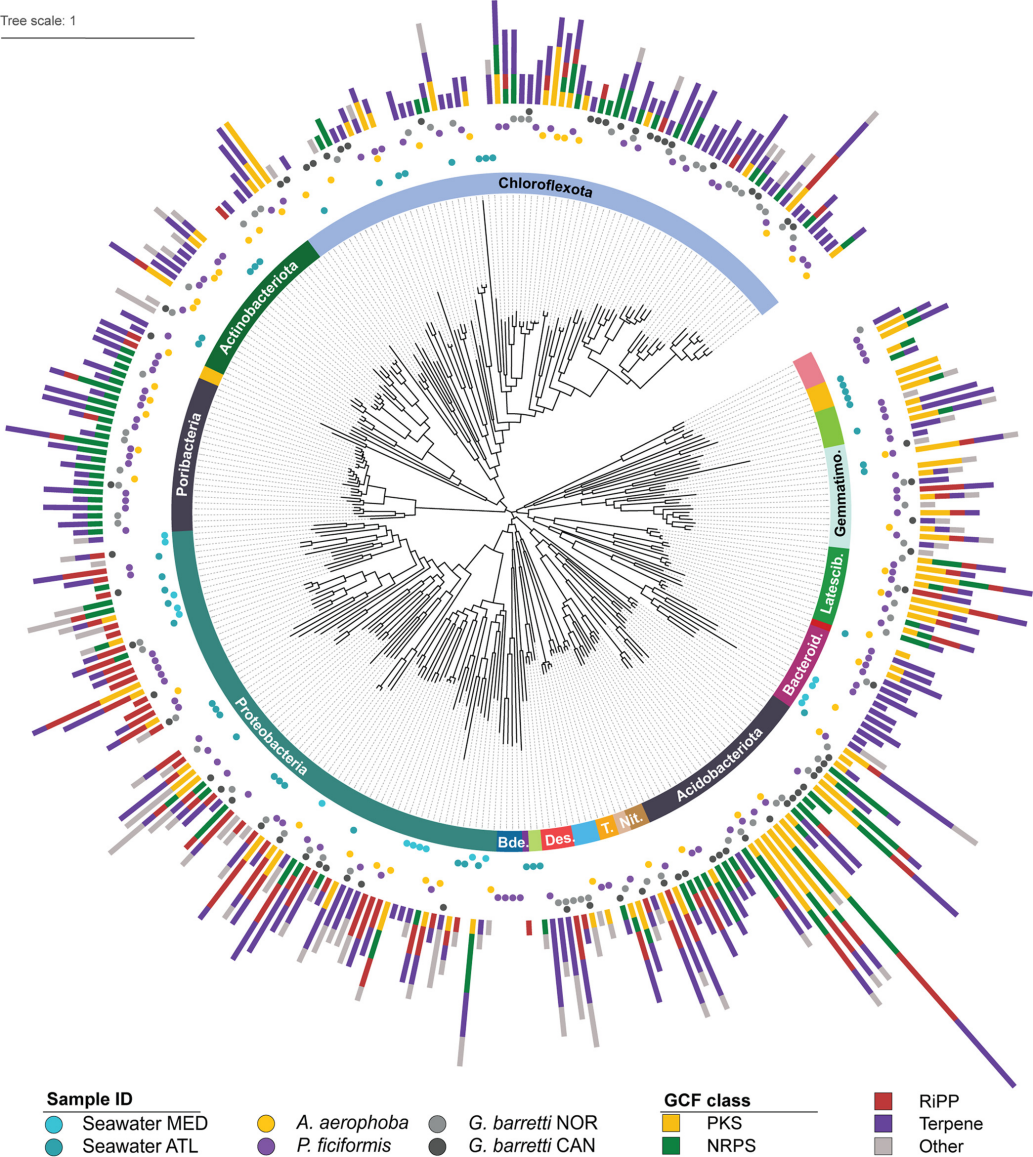
GCFs present in the recovered MAGs. Cladogram based on GTDB classification of MAGs constructed in this study. Layer 1: phylum classification (truncated phyla are *Gemmatimonadota*, *Latescibacterota*, *Bacteroidota*, *Nitrospinota*, *Nitrospinota_A Tectomicrobia*, *Desulfobacterota_B*, and *Bdellovibrionota*). The complete MAG classification is shown in Fig. S2. Layer 2: sample groups where MAGs are observed. Layer 3: GCF content colored by class.

We recovered a total of 316 dereplicated MAGs, all classified at least at the phylum level (GTDB r95), from the sponge and seawater metagenomes (Fig. 4; see also Fig. S2). We observe a relatively low sharedness of MAGs, with only 3% of MAGs being found in more than one sponge species (a MAG is considered shared between samples when MAGs from different samples are clustered by dRep). When the MAGs recovered here are shared, it is mostly so within the same sponge species (*G. barretti* NOR and CAN) or between sponges sharing similar habitats (*A. aerophoba* and *P. ficiformis*). The shared MAGs are widely distributed throughout several phyla (Fig. 4), with *Acidobacteriota* MAGs showing the highest sharedness across species (30% shared MAGs in *A. aerophoba* and *P. ficiformis*). This is in line with the recent work by Robbins et al. (35), who phylogenetically characterized 1,200 MAGs derived from 30 sponge species and reported similar patterns of shared versus exclusive MAGs, with the presence of taxa that are unique to their host sponge species, as well as populations of *Acidobacteriota* that are shared across sponge species. In the specific case of the two *G. barretti* sample groups, we observe that 19% of the MAGs are shared, with proteobacterial MAGs making up the largest fraction (31%) of a set of shared MAGs that nevertheless shows diverse taxonomic assignments (Fig. 4; see also Fig. S2).

10.1128/msystems.00357-22.2 FIG S2 MAG iTOL tree with bin name and taxonomy annotations. Download FIG S2, PDF file, 1.2 MB. Copyright © 2022 Loureiro et al. 2022 Loureiro et al. https://creativecommons.org/licenses/by/4.0/ This content is distributed under the terms of the Creative Commons Attribution 4.0 International license.

Of the 266 MAGs recovered from sponge samples, 96% contained GCFs, and of the 58 MAGs recovered from seawater samples, 83% contained GCFs. In addition, we observe an average of 3.5 GCFs per sponge MAG and 2 GCFs per seawater MAG. *Acidobacteriota* have been known as talented NP producers (42, 54) and also emerge here as a functionally diverse phylum displaying unparalleled biosynthetic potential, represented by 28 MAGs with an average of 6.6 GCFs per MAG. Another diverse and consistently talented phylum is *Latescibacterota* (average 4.4 GCFs per MAG), comprising well-known members of sponge holobiont communities (30, 35, 36) that have only recently been linked to NP production (15) and thus show particular interest for discovery. We observe that members of both these phyla are present in all three sponge species. In addition, two other prolific phyla, yet represented by fewer MAGS, are *Nitrospirota* (average, 8.3 GCFs per MAG) and *Desulfobacterota_B* (average, 6.0 GCFs per MAG). The candidate phylum *Tectomicrobia* is also represented here by three MAGs, respectively encoding 2, 5, and 7 GCFs, and were classified as genus “SXND01” within the family *Entotheonellaceae*. “*Ca.* Tectomicrobia” is famous for its biosynthetically prolific candidate genus *Entotheonella* (25), with other lineages within “*Ca.* Tectomicrobia” currently described as biosynthetically poor (E. Peters, unpublished data). Finally, approximately 40% of all GCFs remained unbinned. This is likely a result of the ineffectivity of current MAG binning methods to successfully process plasmids, mobile elements and genomic islands (55), since BGCs are often located in such mobile regions (56–59).

The previously mentioned SUP (49) and *swf*-like (50) GCFs that were obtained from all sponge species appear to be independent of bacterial taxonomy. They were found in several MAGs of the phyla *Chloroflexota*, *Spirochaetota*, *Proteobacteria*, and *Acidobacteriota* in the case of SUP-like GCFs, and *Nitrospirota*, *Latescibacterota*, and *Acidobacteriota* in the case of *swf*-like GCFs. Multiple MAGs encoding SUP and *swf*-like GCFs belonging to different phyla were observed within the same sponge holobiont. However, despite the similarity of taxa harboring the SUP-like and *swf*-like GCFs at the phylum level, at the species level (<95% gANI) the majority of these MAGs are specific to a single sponge species (see Table S4).

10.1128/msystems.00357-22.7 TABLE S4 SUP-like and *swf*-like GCF examples binned in MAGs, characterized by MAG distribution and phylum taxonomy. Download Table S4, PDF file, 0.2 MB. Copyright © 2022 Loureiro et al. 2022 Loureiro et al. https://creativecommons.org/licenses/by/4.0/ This content is distributed under the terms of the Creative Commons Attribution 4.0 International license.

### Widespread ether-lipid-associated BGCs in sponges.

**FIG 5:**
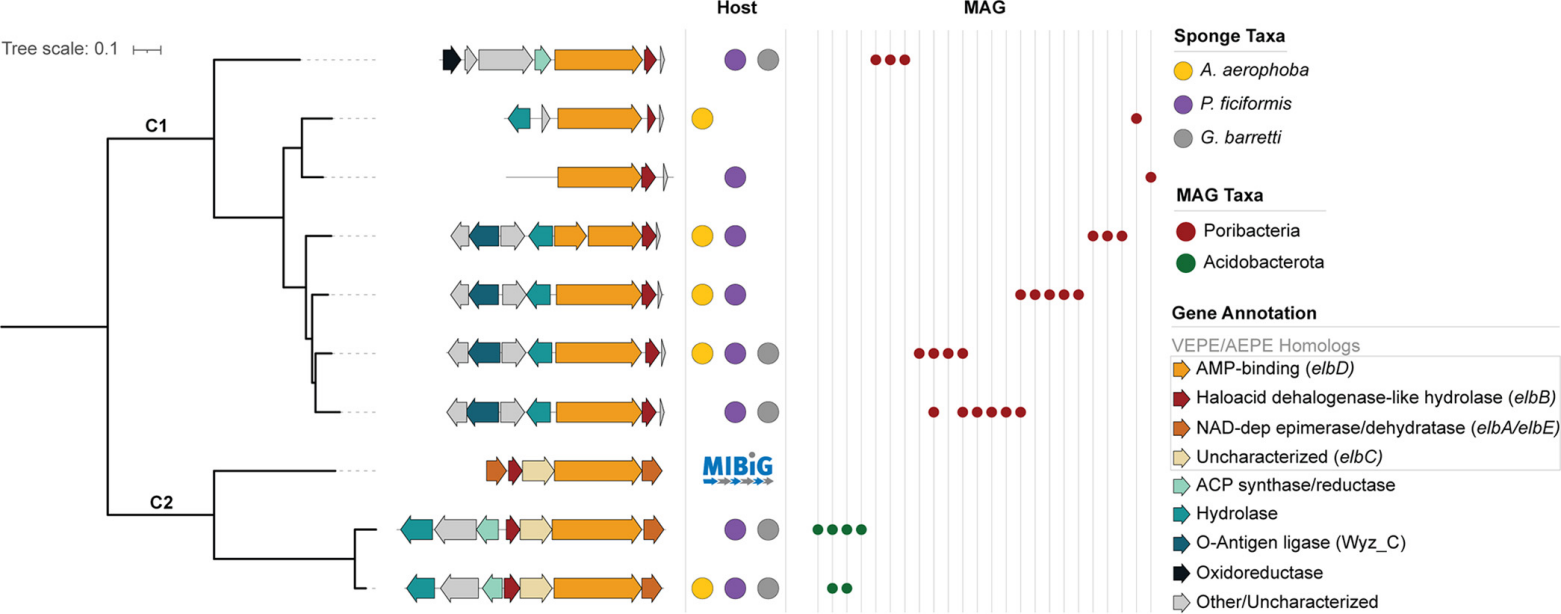
Distribution and characterization of the VEPE-related GCFs. The FastTree phylogeny of VEPE-associated GCFs, each characterized by a representative BGC, based on sequence similarity of the A-domain (AMP-binding), is shown. A detailed overview of these GCFs is provided in Fig. S3. C1 and C2 refer to clades 1 and 2. Genes are colored based on predicted function. Each BGC is annotated by its presence in host sponge species and in MAGs.

Within the shared GCF core, we identified nine nonribosomal peptide synthetase (NRPS)-like GCFs with a similar architecture: an NRPS-like core gene (*elbD* homolog) containing fatty acyl coenzyme A (acyl-CoA)-like reductase, acyl-CoA synthetase, thiolation, and acylglycerolphosphate acyltransferase domains. The NRPS-like core gene was consistently flanked by hydrolases/dehydratases, as well as oxidoreductases and genes involved in fatty acid biosynthesis (Fig. 5). These GCFs show similarity to a known BGC encoding the production of vinyl-/alkyl-ether lipids (VEPE/AEPE; MiBIG accession no. BGC0000871). The VEPE/AEPE lipids are produced by Myxococcus xanthus DK 1622 as extracellular signals guiding fruiting body morphogenesis and sporulation (46, 60). It is postulated that these lipids are generated via modification of phospholipids originating from the cell membrane with participation of BGC0000871’s genes *elbB*, *elbD*, and *elbE*, as well as the additional desaturase *carF* (60, 61). An *elbD* homolog of poribacterial origin has also been identified by Lorenzen et al. (46).

10.1128/msystems.00357-22.3 FIG S3 Detailed depiction of BGC0000871-like GCFs captured by CORASON and included in NRPS-like ether lipid analysis. GCFs are numbered, and the red box indicates the chosen representative. Download FIG S3, PDF file, 0.7 MB. Copyright © 2022 Loureiro et al. 2022 Loureiro et al. https://creativecommons.org/licenses/by/4.0/ This content is distributed under the terms of the Creative Commons Attribution 4.0 International license.

We observe architectural changes in the adjacent genes of the cluster across the set that are congruent with the clades generated based on sequence similarity of the A domain (Fig. 5). Additional analysis of adenylation domain active site specificity-conferring residues (AdenylPred [62]; see Table S5) indicates potentially different functional classes and substrate specificities for the two clades, which suggests diversity in the chemical compounds produced by the encoded machinery. Gene clusters from the two clades are associated with distinct bacterial taxa, being specific to *Acidobacteriota* and *Poribacteria* (GTDB r95) MAGs, with any given MAG harboring a maximum of two of the nine GCFs (see Table S6).

10.1128/msystems.00357-22.8 TABLE S5 Ether lipid A domain AdenylPred predictions, C1 and C2 refer to clades 1 and 2 as in Fig. 5. Prediction probabilities are averaged within each clade. Download Table S5, PDF file, 0.3 MB. Copyright © 2022 Loureiro et al. 2022 Loureiro et al. https://creativecommons.org/licenses/by/4.0/ This content is distributed under the terms of the Creative Commons Attribution 4.0 International license.

10.1128/msystems.00357-22.9 TABLE S6 MAGs containing ether lipid GCFs, and characterized by phylum taxonomy. Download Table S6, PDF file, 0.5 MB. Copyright © 2022 Loureiro et al. 2022 Loureiro et al. https://creativecommons.org/licenses/by/4.0/ This content is distributed under the terms of the Creative Commons Attribution 4.0 International license.

Ether lipids seen in the sponge holobiont are often linked to pathogen defense by showing antimicrobial activity (63–66). However, the biosynthetic origin and pathway for these molecules is currently undescribed. Ultimately experimental work will be necessary to determine the function and biosynthesis of these ether lipids within the sponge holobiont.

### High RiPP diversity in sponge bacterial symbionts.

**FIG 6:**
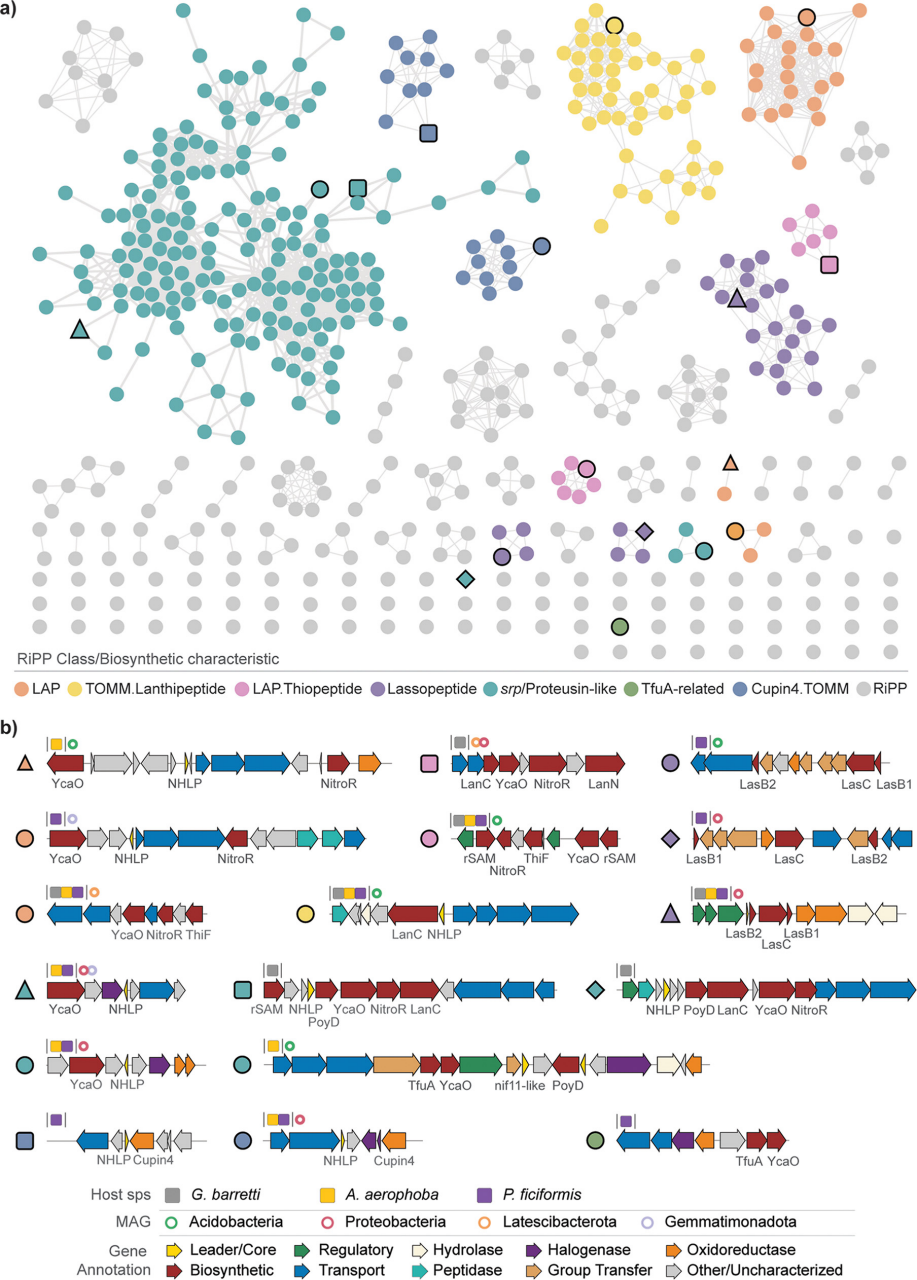
RiPP diversity. (a) RiPP BGCs represented in a BiG-SCAPE-generated similarity network. Highlighted nodes are colored by biosynthetic class/characteristic, boldface node shapes link to the specific BGCs described below, and gray nodes represent nonhighlighted RiPP BCGs. (b) Representative BGCs from each highlighted GCF are depicted and characterized by the predicted class, the presence of GCF in a host sponge, and MAG (GTDB r95). Genes are colored by predicted function.

Even though the sponge holobiont is recognized as an extensive source of diverse NPs, its inventory of ribosomally synthesized and posttranslationally modified peptides (RiPPs) has remained largely undescribed, with one exception being the proteusin polytheonamides (15, 25, 67). Here, we expand the known repertoire in sponge holobionts and showcase sponges’ RiPP diversity by identifying 17 uncharacterized RiPP families which seem to be widespread in sponge holobionts. In some of these cases, GCFs are recovered from all sponge species, which points toward an important role of the produced NPs in the context of sponge-microbe symbiosis (Fig. 6).

RiPPs constitute a class of natural products that is produced from a ribosomally synthesized precursor peptide, composed of an N-terminal leader and a C-terminal core, which is modified by biosynthetic enzymes encoded in the BGC, and matured in a final proteolytic cleavage step (68). We see a high incidence of RiPP BGCs with predicted nitrile hydratase-like leader peptide (NHLP) domains, which have been identified as proteusin precursor peptides, as well as nif11-like leader peptide domains associated with thiazole/oxazole-modified microcins (TOMM) precursor peptides (69). Encoded within these BGCs are a number of modifying enzymes that span several RiPP subclasses: YcaO cyclodehydratases (70, 71), radical *S*-adenosylmethionine (rSAM) enzymes (72) including PoyD (67), lanthionine synthetase C-like (LanC) enzymes (73), nitroreductases (NitroR) (74), and TfuA-like enzymes (75). Other modifying enzymes include oxidoreductases, group transferases, and halogenases. Several RiPP subclasses contain YcaO-generated azol(in)e heterocycles (71), such as the linear azol(in)e-containing peptides (LAP) and thiopeptides, and are often linked to pharmacologically interesting bioactivities (76–78). The potential for azol(in)e-containing RiPPs has been found widespread in bacterial genomes (79) but has only recently been reported in marine sponge microbiomes with the identification of the *srp* clusters by Nguyen et al. (15). We predict an additional structurally diversified set of azol(in)e-containing RiPPs to this repertoire, expanding the combinatorial space of modifying enzymes and hence the potential for production of bioactive NPs.

In addition, we observed four GFCs that harbor a cupin-4 modifying enzyme in addition to an NHLP domain, which has also been connected to proteusin biosynthesis (80). The cupin superfamily is functionally highly diverse, with reported hydroxylation, epoxidation, dioxygenation, decarboxylation, dehydration, and halogenation activities (81–83). Although these genes appear in marine environments, they are not yet regarded as a typical constituent of the sponge microbiome biosynthetic array (84, 85). We further identified several GCFs predicted to be responsible for the production of highly modified lassopeptides, encoding additional hydrolases, oxidoreductases, and group transferases (sulfo-, glycol-, and nucletotidyl-). These include GCFs that are shared among all sponge species. Lassopeptides have been found in some sponge-associated microbiomes (86, 87), but their presence together with diverse flanking enzyme-encoding genes has not been previously reported in this environmental niche. While the functions of these BGCs and their metabolic products remains elusive, this data set expands and diversifies the set of sponge-holobiont-encoded RiPPs and predicts sponge microbiomes to be prolific sources of RiPP NPs.

### Conclusion.

Here, we describe a systematic analysis of the specialized metabolite diversity landscape of several marine sponge holobionts, whose metagenomes contain a large number of uncharacterized BGCs. We show that while there is high biosynthetic diversity that is unique to each sponge species’ symbiotic community, a small functional core is conserved throughout the studied sponge species. This functional core includes a novel group of NRPS-like ether lipid-associated GCFs widespread across sponges and a number of RiPPs. We recovered a consistent set of biosynthetically potent MAGs from the metagenomes, with *Acidobacteriota* and *Latescibacterota* standing out as potential prolific NP producers. This study thus contributes to a growing body of research uncovering the network of NPs with elusive functions that are generated by marine sponge microbiomes.

## MATERIALS AND METHODS

### Sponge collection.

Norway *Geodia barretti* (*G. barretti* NOR) samples (gb1-gb10) were collected and processed by E. Peters et al. (unpublished data), and Canada *Geodia barretti* (*G. barretti* CAN) samples (gb126, gb278, gb305) were collected and processed by Steffen et al. (107). Atlantic Seawater (Seawater ATL) samples (gb1_f – gb10_f) were collected onboard R/V Hans Brattström of the University of Bergen from Korsfjord, Bergen, Norway (60°8.13′N, 5°6.7′E) in September and October 2017 by filtering 2 L of seawater through polyvinylidene difluoride membrane filters (pore size, 0.22 μm; diameter, 47 mm; Merck Millipore, Burlington, MA). Filters were snap-frozen in liquid nitrogen and stored at −80°C. *Aplysina aerophoba* and Mediterranean Seawater (Seawater_MED) samples were collected and processed as described by Chaib De Mares et al. (88) and are publicly available (88). *Petrosia ficiformis* sampling took place in August 2018 at a semisubmerged marine cave (5- to 6-m depth) with internal freshwater springs in Sfakia, Greece (35°12′N, 24°7′E) in a collaborative effort with the Hellenic Centre for Marine Research (HCMR). Immediately after collection, the samples were transferred to liquid nitrogen and stored at −80°C.

### Total DNA extraction and metagenomic sequencing.

*A. aerophoba*, *G. barretti*, and *P. ficiformis* sponge samples were crushed in liquid nitrogen to a fine power with pestle and mortar. A 200-mg portion of sponge tissue powder was further disrupted by bead beating using milling balls (5 × 2 mm + 2 × 5 mm), followed by two steps of shaking for 20 s at 4,000 rpm in a Precellys 24 tissue homogenizer (Bertin Instruments, Montigny-le-Bretonneux, France) (89). Tissue lysate was further used for DNA extraction with an AllPrep DNA/RNA/protein minikit (Qiagen, Hilden, Germany). Total DNA extraction of seawater filters was done using the entire filter, following the protocol described above. DNA extracted from water filter replicates gb5_f and gb6_f was pooled prior to sequencing to meet minimum DNA quantity requirements. The extracted DNA was further cleaned by a collagenase treatment using C9891 collagenase from Clostridium histolyticum (Sigma-Aldrich, St. Louis, MO) at a concentration of 2.5 mg/mL for 30 min at 4°C with vortexing every 5 min at max speed for 10 s. It was then purified using a MasterPure Gram-positive DNA purification kit (Lucigen, Middleton, WI) according to the manufacturers’ instructions and passed through Illustra MicroSpin S-400 HR columns (GE Healthcare, Chicago, IL). *G. barretti*, *P. ficiformis*, and Seawater_ATL total DNA was obtained; this was sequenced by Novogene (Hong Kong, China) using an Illumina HiSeq PE150 platform. *A. aerophoba* and Seawater_MED total DNA was sequenced by Research Group Genome Analytics (GMAK) at the DSMZ (Braunschweig, Germany) using Illumina HiSeq PE100. *A. aerophoba* DNA was also prepared for Pacific Biosciences (PacBio, Menlo Park, CA) long-read sequencing and was processed as described previously (90) and sequenced at the DSMZ. Complete sample metadata can be found in Table S1 in the supplemental material.

10.1128/msystems.00357-22.4 TABLE S1 Sample metadata. Download Table S1, PDF file, 0.2 MB. Copyright © 2022 Loureiro et al. 2022 Loureiro et al. https://creativecommons.org/licenses/by/4.0/ This content is distributed under the terms of the Creative Commons Attribution 4.0 International license.

### Quality trimming and adapter removal.

Illumina HiSeq read adapter removal, quality filtering and normalization was done using the BBduk.sh script from the BBTools suite v37.64 (91), following user guide instructions, with the parameters ktrim=r k=23 mink=7 hdist=1 tpe tbo qtrim=rl trimq=20 ftm=5 maq=20. The minlen parameter was set to 30 for *A. aerophoba* and Med_SW samples, and to 50 for all remaining samples. BBDuk (91) was also used to remove sequencing artifacts and phi X contamination, with default settings.

### Metagenomic assembly.

Reads were normalized for coverage with BBNorm (91) with the parameters target=100 min=5 for *Petrosia ficiformis* (sequenced and processed at a later stage), and target=200 min=3 for all other samples. As SPAdes v3.12 (92) hybrid mode (–pacbio) does not support coassembly, *A. aerophoba* sample Aply22 sequencing replicates were merged prior to hybrid assembly. *A. aerophoba* filtered Illumina HiSeq reads and PacBio reads were assembled with SPAdes v3.12 (92) using the –meta and –only-assembler flags. Filtered Illumina HiSeq reads from all other samples were assembled with SPAdes v3.12 (92) using the –meta and –only-assembler flags.

### MAG binning, dereplication, and classification.

Contigs were binned using metaWRAP v1.2 (93) with minimum completeness of 75% and maximum contamination of 10% (see Table S3), using MaxBin2 (94), metaBAT2 (95), and CONCOCT (96). The obtained bins were dereplicated using dRep (97) v2.5.4 with default parameters for primary clustering and secondary clustering using parameters –S_algorithm gANI –S_ani 0.95 (98). The dereplicated bins were taxonomically classified using the GTDB-Toolkit (99) v1.1.0 (GTDB-Tk) classify workflow. A phylogenetic tree of the dereplicated bins was created using the multiple sequence alignment generated in the GTDB-Tk (99) align workflow with FastTree (100) v2.1.11, default parameters. This tree was visualized and annotated using the Interactive Tree of Life (101) (iTOL v6) online tool.

10.1128/msystems.00357-22.6 TABLE S3 MAG completeness and contamination. Download Table S3, PDF file, 0.1 MB. Copyright © 2022 Loureiro et al. 2022 Loureiro et al. https://creativecommons.org/licenses/by/4.0/ This content is distributed under the terms of the Creative Commons Attribution 4.0 International license.

### Metagenome 16S rRNA gene taxonomic classification.

16S rRNA gene sequences were extracted and characterized using Phyloflash (102) v3 with the SILVA rRNA gene database (103) v38.1, the parameters -taxlevel 6 -poscov and -readlength 150 for *G. barretti*, Seawater_Atl and *P. ficiformis* samples, 100 for *A. aerophoba* and Seawater_Med Taxa relative abundance plots (–task barplot –level 6), and an NTU table (–task ntu_table –level 6) were generated with phyloFlash_compare.pl.

### Biosynthetic gene cluster analysis.

BGC prediction was performed for all contigs over 4000 bp in length using antiSMASH (40) v5 using the following parameters: –cb-general –cb-subclusters –cb-knownclusters –minlength 4000 –hmmdetection-strictness relaxed –genefinding-tool prodigal-m –clusterhmmer –asf –smcog-trees –pfam2go. BiG-SCAPE (41) v1.0.1 was run on all predicted BGCs using parameters –mix -v –mode auto –mibig –cutoffs 0.5 –include_singletons. BiG-SCAPE (41) network files were processed by in-house Python scripts available at https://github.com/CatarinaCarolina/sponge_meta_BGC to generate Fig. 1a, b, and e, as well as Fig. 5, using the Python package UpSetPlot v0.4.1. Phylogenetic analysis of NRPS-like GCFs was conducted using CORASON (41) v1, default parameters, with gb8_2 contig 859 gene 9 as a query and MIBiG BGC0000871.1 as reference BGC, and nine GCFs were selected for further analysis (see Fig. S3). A representative BGC was selected from each of these, based on BGC completeness and best representation of adjacent gene diversity encoded in the GCF. The respective AMP domain amino acid sequences were used in a multiple sequence alignment (MSA) done with Muscle (104) v3.8.31. FastTree (100) v2.1.11 processed the MSA into a phylogenetic tree, visualized with iTOL (101). A RiPP-specific BiG-SCAPE (41) run was carried out by selecting all RiPP BGCs previously identified, excluding those classified with the antiSMASH (40) rules DUF692 and TIGR03975, since these BGCs were likely false positives based on manual inspection. GCF abundance, i.e., normalized RPKM (reads per kilobase per million), was calculated using BiG-MAP (105) with parameters -tg 0 -c 0.5 for the family module, and otherwise at default settings.

### Sequence estimated coverage.

Sample read redundancy estimation was calculated using Nonpareil (106) v3.304 with parameters -T kmer -X 1000000. Nonpareil curves were built using the R package Nonpareil in RStudio, R v4.0.3.

### Statistical and diversity analysis.

The Phyloflash (101) bacterial/16S-rRNA-gene-derived NTU table, normalized by relative abundance, and the BiG-MAP (105) normalized RPKM table were used to calculate Sample Shannon alpha-diversity scores with the Python package skbio.diversity.alpha.shannon, as well as to generate a Bray-Curtis dissimilarity matrix with the Python package scipy.spatial.distance.braycurtis. Pearson’s correlation score for Shannon alpha-diversity scores was calculated using the Python package scipy.stats.pearsonr. A principal coordinate analysis using these same distance matrices was performed using the Python package skbio.stats.ordination.pcoa. ERMANOVA was calculated for both distance matrices grouping by sample type using the Python package skbio.stats.distance.permanova. Python scripts used to carry out this analysis are available at https://github.com/CatarinaCarolina/sponge_meta_BGC.

### MAG and GCF data integration.

MAG iTOL (101) annotation tables were generating by processing GTDB-Tk and BiG-SCAPE outputs with in-house Python scripts available at https://github.com/CatarinaCarolina/sponge_meta_BGC.

### Data availability.

The data for this study have been deposited in the European Nucleotide Archive (ENA) at EMBL-EBI under accession number PRJEB51534. Python scripts created for this analysis are available (https://github.com/CatarinaCarolina/sponge_meta_BGC).
