# Supplementary figures and images for: Comparative Metagenomic Analysis of Biosynthetic Diversity across Sponge Microbiomes Highlights Metabolic Novelty, Conservation, and Diversification
Source: mSystems. 2022 Jul 18;7(4):e00357-22. doi: 10.1128/msystems.00357-22 (PMC9426513; doi:10.1128/msystems.00357-22)

Figure S1

a)

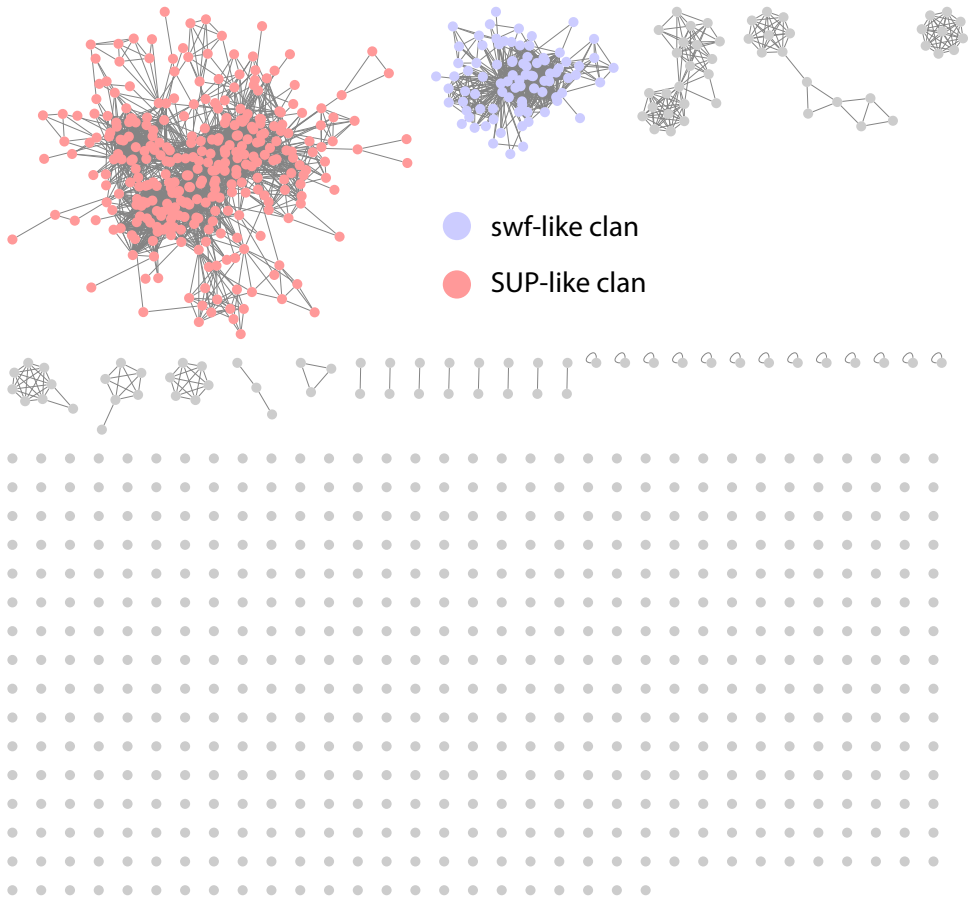

b)

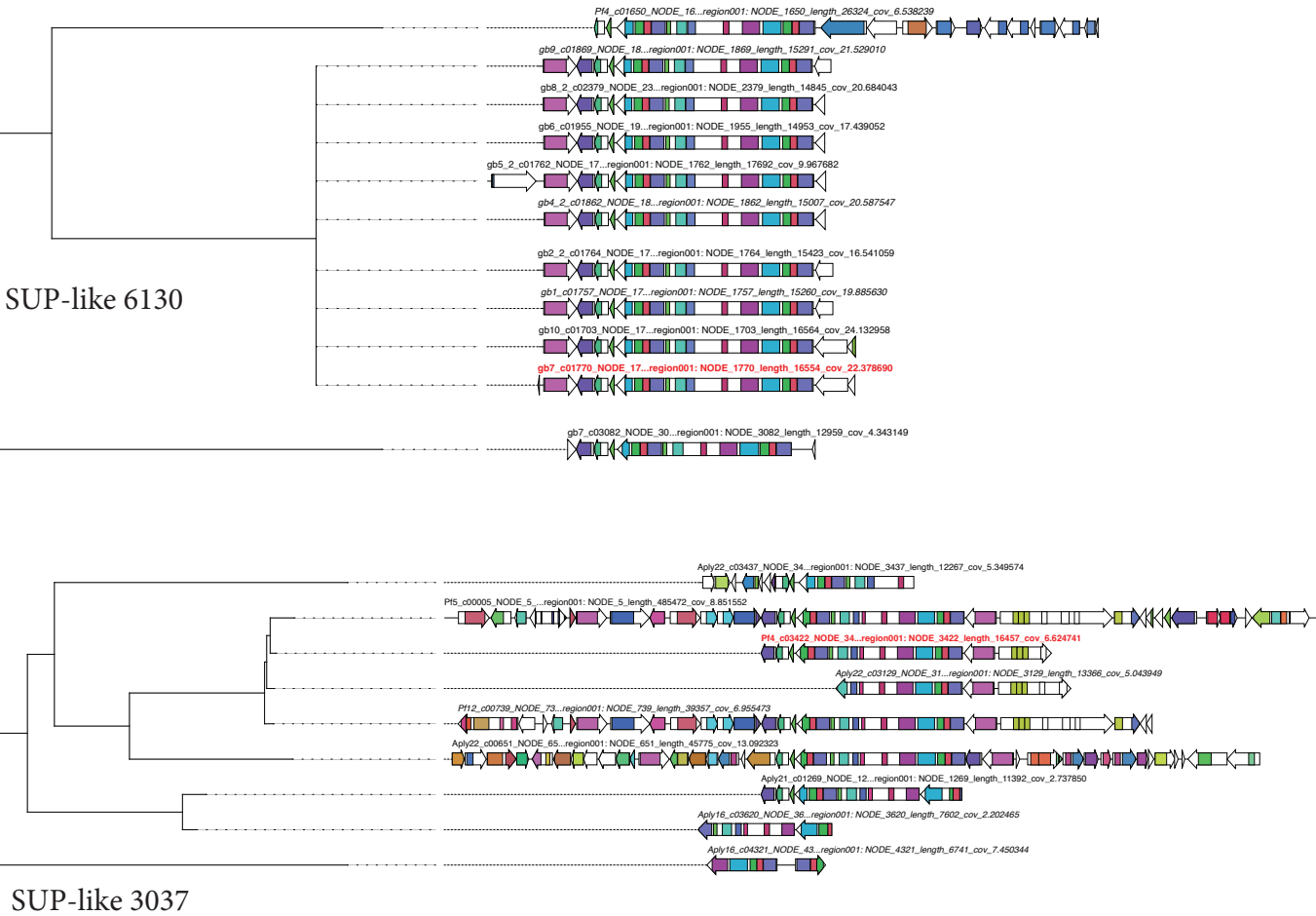

swf-like 2399

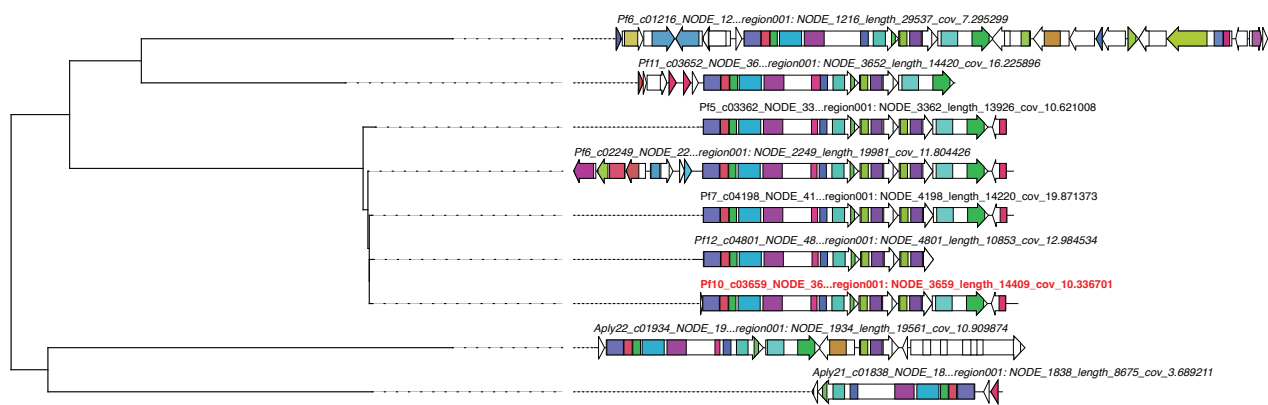

swf-like 6055

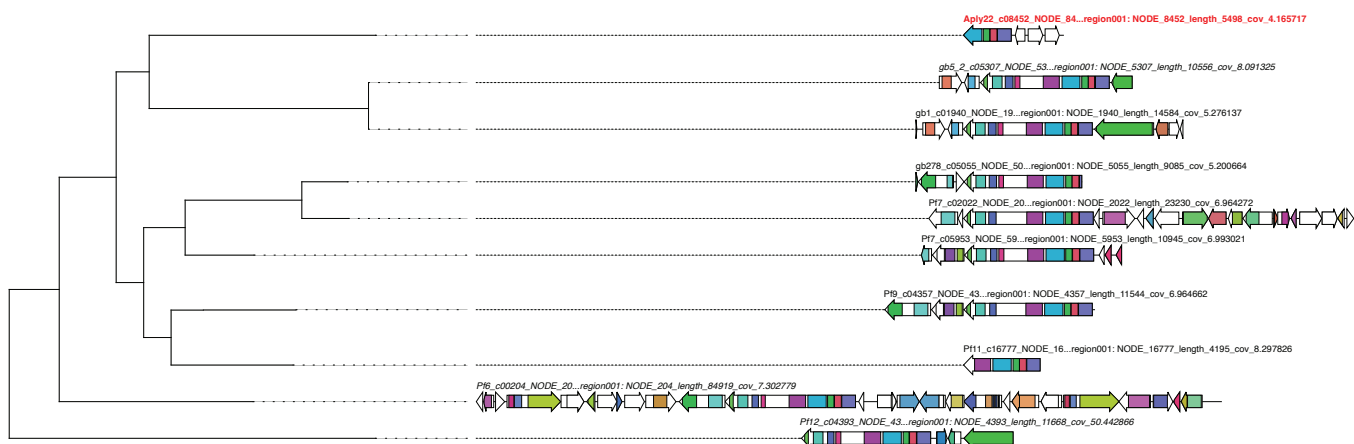

Supplement: FIG S1 [file msystems.00357-22-s0001.pdf]

Figure S2

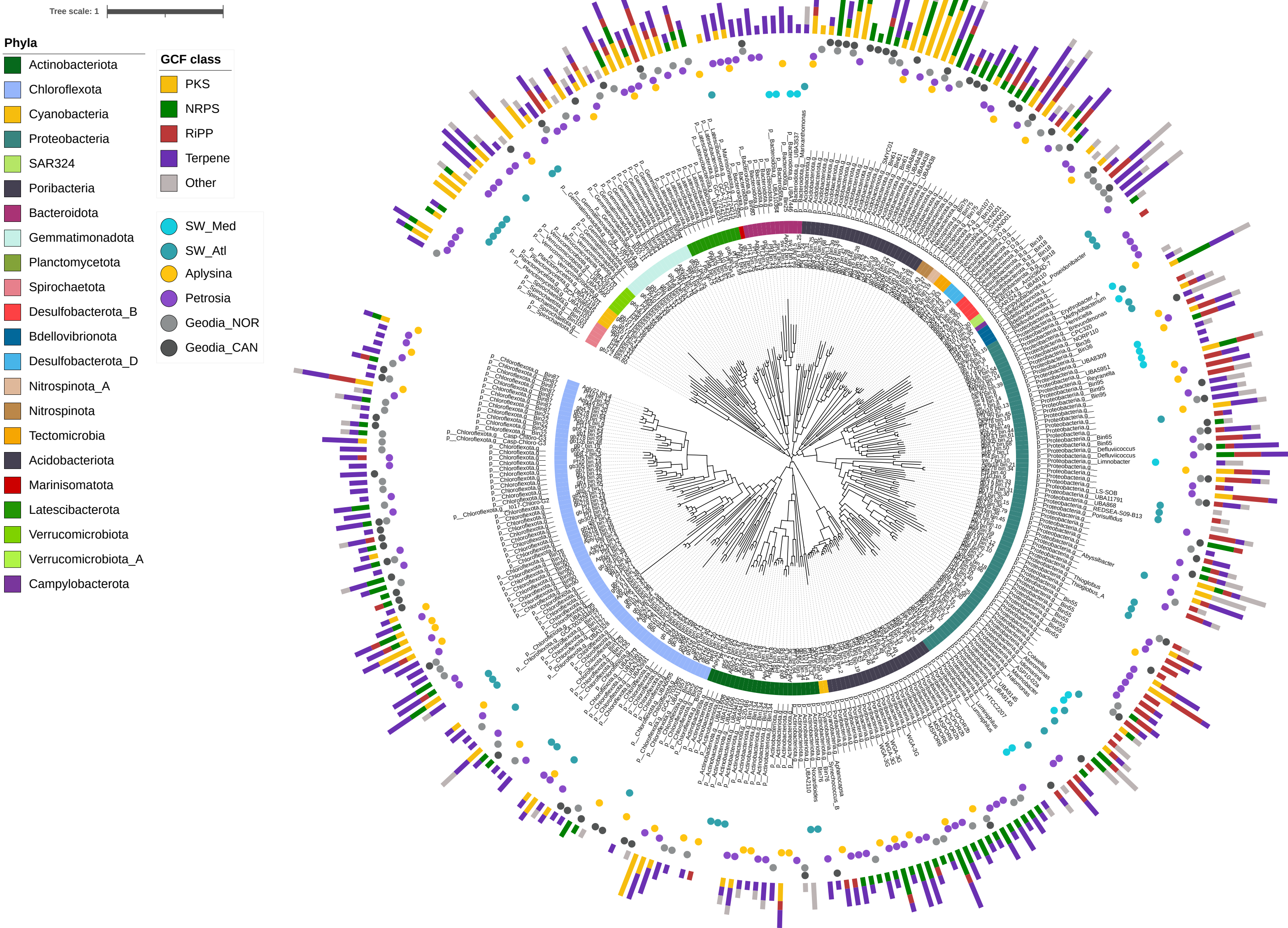

Supplement: FIG S2 [file msystems.00357-22-s0002.pdf]

Figure S3

5496

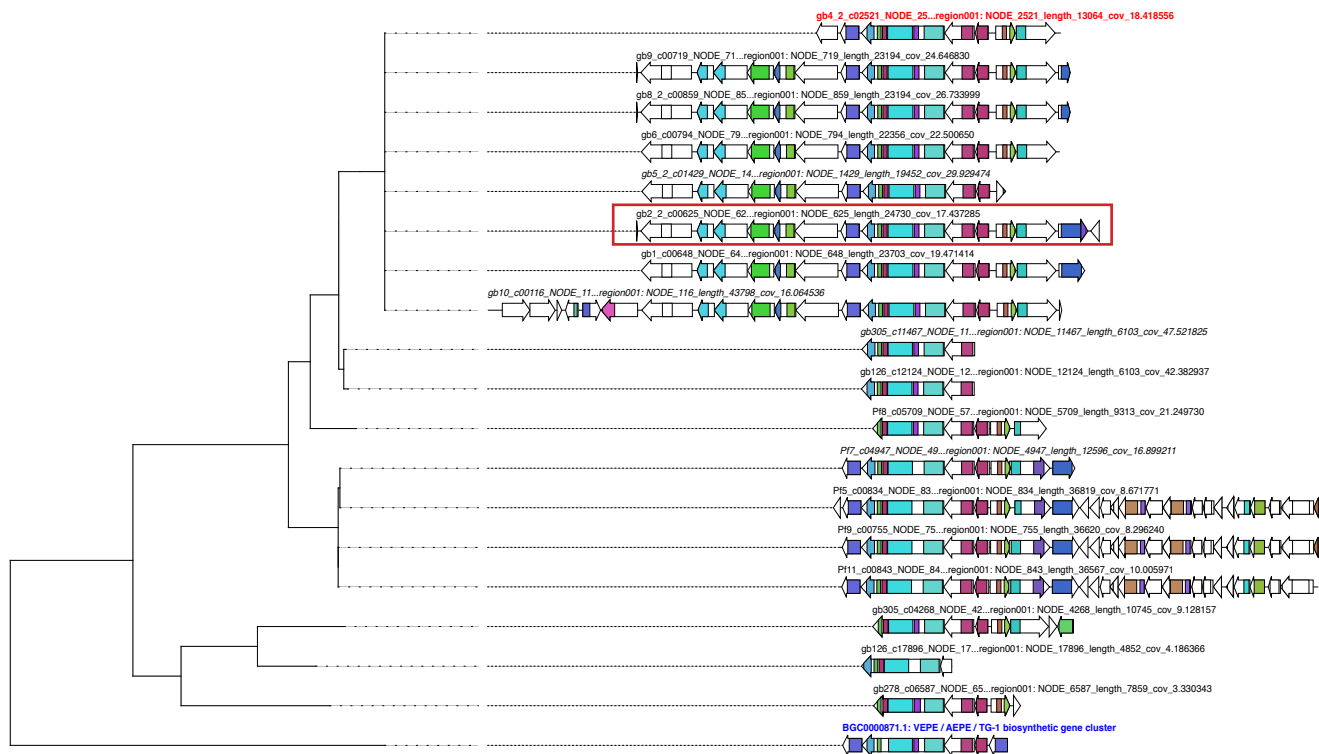

380

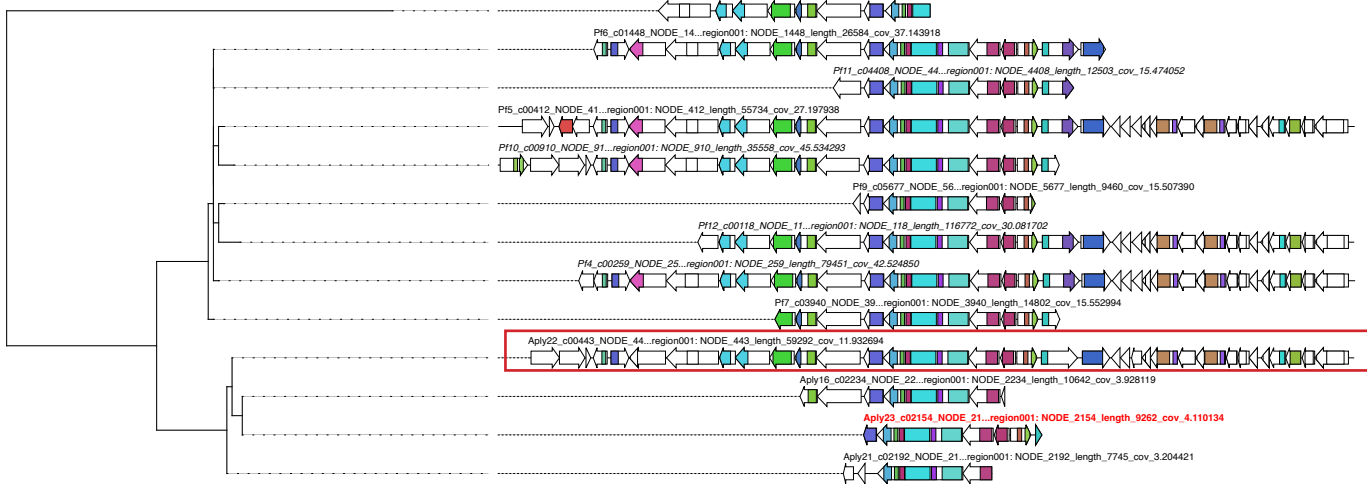

3574

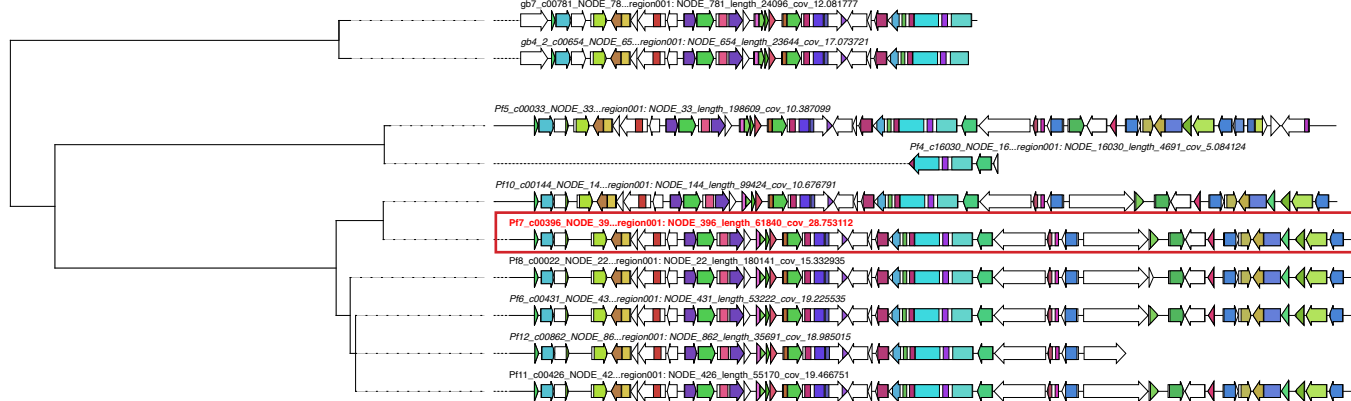

6120

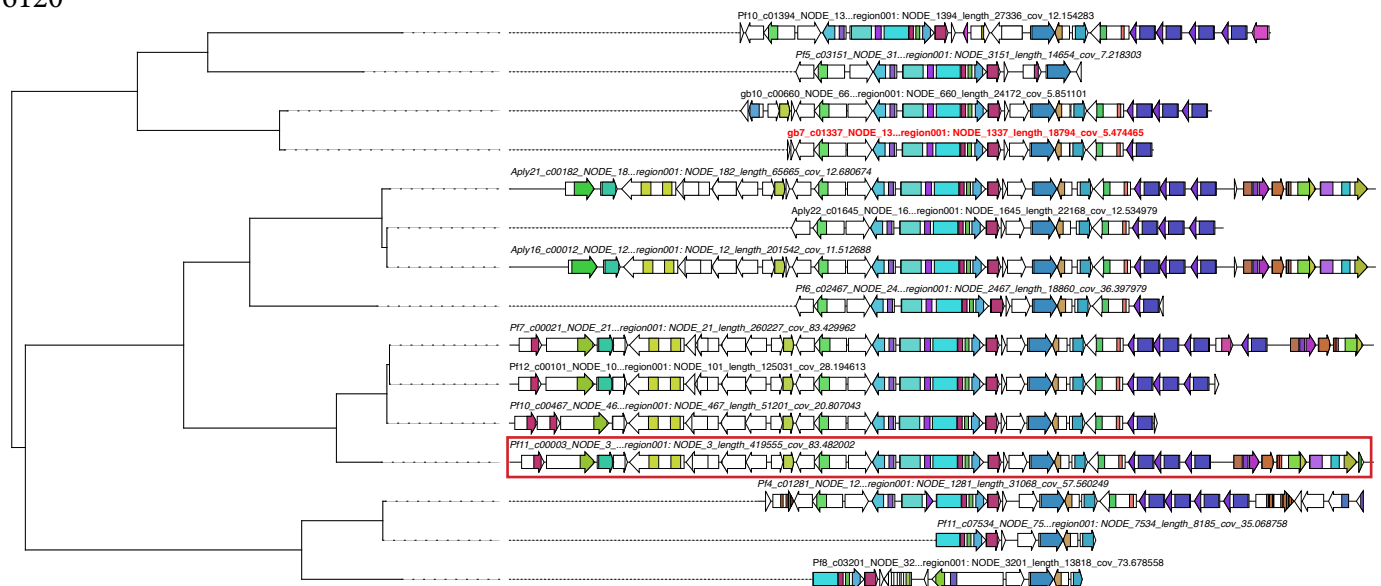

4988

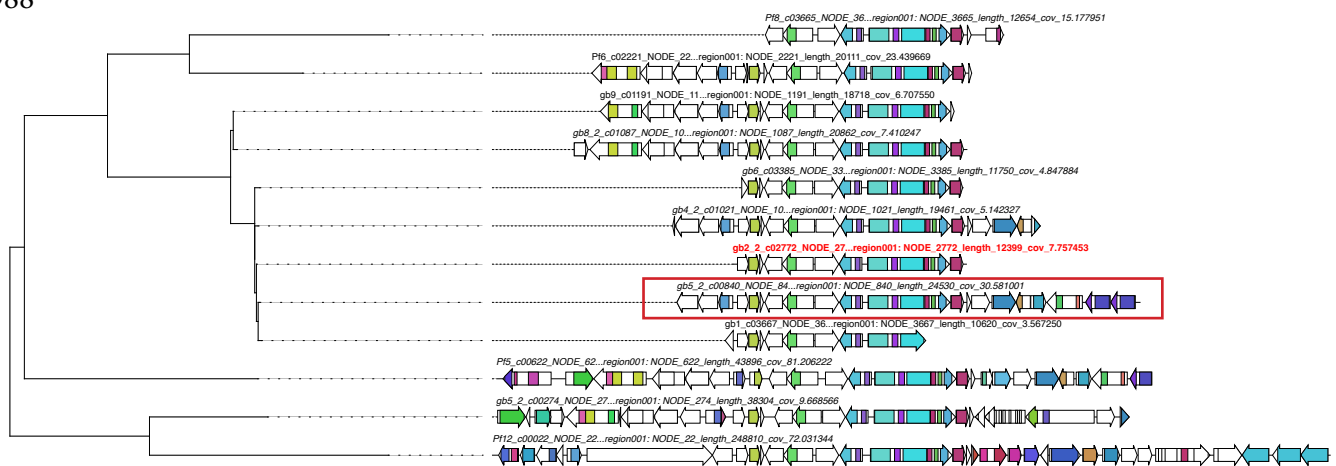

3047

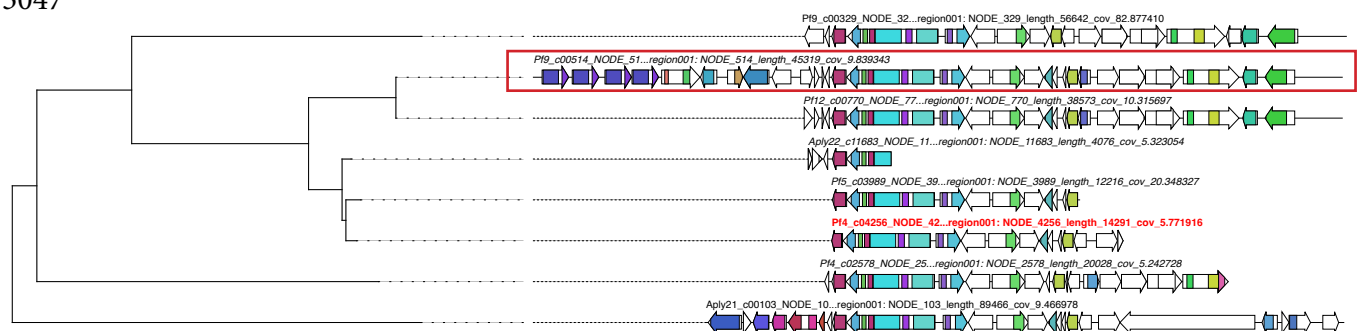

4093

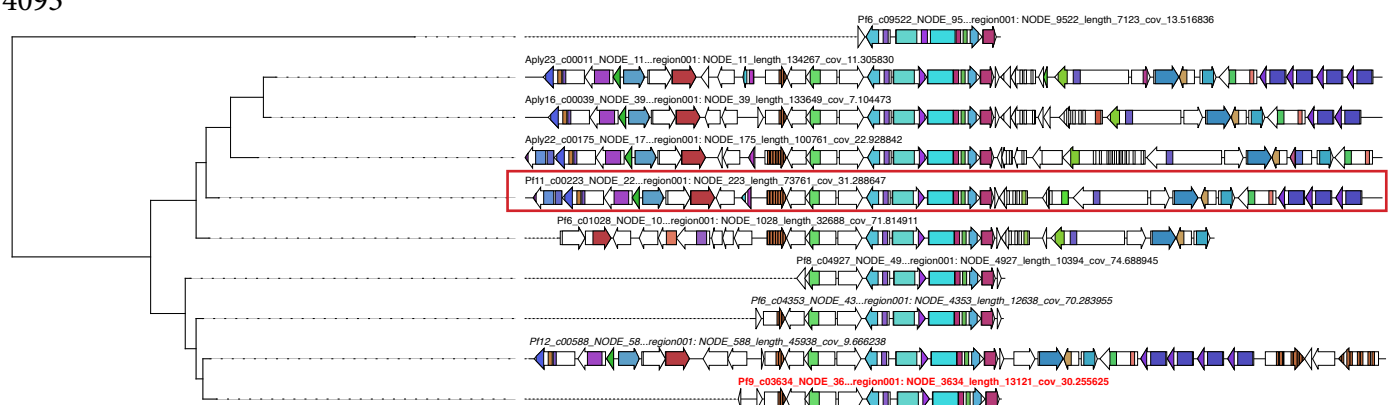

172

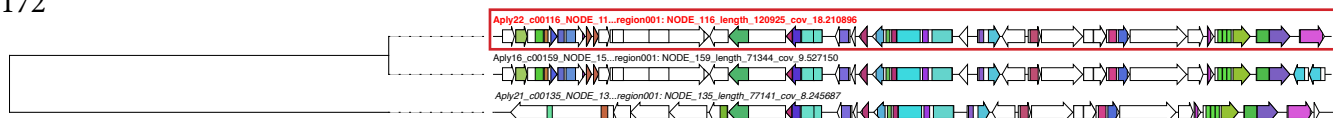

4034

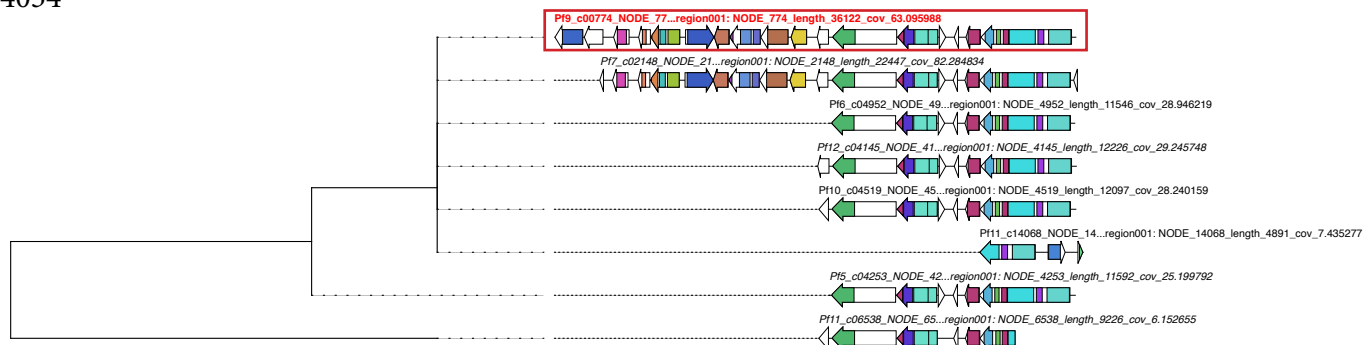

Supplement: FIG S3 [file msystems.00357-22-s0003.pdf]
