# Supplementary material for: Comparative Metagenomic Analysis of Biosynthetic Diversity across Sponge Microbiomes Highlights Metabolic Novelty, Conservation, and Diversification
Source: mSystems. 2022 Jul 18;7(4):e00357-22. doi: 10.1128/msystems.00357-22 (PMC9426513; doi:10.1128/msystems.00357-22)
Supplement: TABLE S2 [file msystems.00357-22-s0005.pdf]

Table S2

PERMANOVA Results	Taxonomy - based	GCF - based
Input	phyloFlash_compare.6.ntu_table.braycurtis	BiG_MAP.all_RPKM_NORM.braycurtis
test statistic name	pseudo-F	pseudo-F
sample size	35	35
number of groups	6	6
test statistic	55.7199	97.4049
p-value	0.001	0.002
number of permutations	999	1000

	GCF-based alpha diversity scores			Taxonomy-based alpha diversity scores		
	Mean	Standard Deviation	Variance	Mean	Standard Deviation	Variance
All-Sponges	8.45	0.13	0.02	5.99	0.26	0.07
G. barretti NOR	8.36	0.07	0.01	5.71	0.08	0.01
G. barretti CAN	8.29	0.10	0.01	6.13	0.11	0.01
P. ficiformis	8.59	0.05	0.01	6.09	0.10	0.01
A. aerophoba	8.46	0.08	0.01	6.36	0.17	0.03
All-Seawater	6.94	0.74	0.56	6.96	0.16	0.03

	GCF-based		Taxonomy-based	
	Kruskal-Wallis' H	pvalue	Kruskal-Wallis' H	pvalue
All-Sponges	17.10	0.0007	19.97	0.0002
G. barretti NOR & CAN	1.029	0.31	6.43	0.011
G. barretti (NOR&CAN) & *A. aerophoba*	3.92	0.048	8.0	0.005
A. aerophoba & *P. ficiformis*	4.67	0.031	5.36	0.021
G. barretti (NOR&CAN) & *P. ficiformis*	12.33	0.0005	13.5	0.0002

All-Sponges Coefficient of Determination = -18.64
All-Sponges Spearman r = -0.4159663865546219, pvalue = 0.013
