# Supplementary material for: Comparative Metagenomic Analysis of Biosynthetic Diversity across Sponge Microbiomes Highlights Metabolic Novelty, Conservation, and Diversification
Source: mSystems. 2022 Jul 18;7(4):e00357-22. doi: 10.1128/msystems.00357-22 (PMC9426513; doi:10.1128/msystems.00357-22)
Supplement: TABLE S4 [file msystems.00357-22-s0007.pdf]

Table S4

**SUP-like example**		
GCF	Sponge species	
**6130**	**Aplysina,Petrosia,Geodia**	
Encoding bin	Bin presence host	Bin taxonomy
Pf4_bin.62.fa	Petrosia	p__Chloroflexota
Pf7_bin.11.fa	Petrosia	p__Spirochaetota
Pf9_bin.9.fa	Petrosia	p__Proteobacteria
Pf8_bin.5.fa	Petrosia	p__Proteobacteria
Pf6_bin.10.fa	Petrosia	p__Proteobacteria
Pf5_bin.39.fa	Petrosia	p__Acidobacteriota
Pf8_bin.46.fa	Aplysina,Petrosia	p__Acidobacteriota
gb5_2_bin.68.fa	Geodia	p__Acidobacteriota

**swf-like example**		
GCF	Sponge species	
**2399**	**Aplysina, Petrosia**	
Encoding bin	Bin presence in host	Bin taxonomy
Aply22_bin.37.fa	Aplysina	p__Nitrospirota
Pf7_bin.67.fa	Aplysina, Petrosia	p__Latescibacterota
Pf10_bin.11.fa	Petrosia	p__Acidobacteriota

**swf-like example**		
GCF	Sponge species	
**6055**	**Aplysina,Petrosia,Geodia**	
Encoding bin	Bin presence in host	Bin taxonomy
gb10_bin.62.fa	Geodia	p__Latescibacterota
