# Supplementary material for: Comparative Metagenomic Analysis of Biosynthetic Diversity across Sponge Microbiomes Highlights Metabolic Novelty, Conservation, and Diversification
Source: mSystems. 2022 Jul 18;7(4):e00357-22. doi: 10.1128/msystems.00357-22 (PMC9426513; doi:10.1128/msystems.00357-22)
Supplement: TABLE S5 [file msystems.00357-22-s0008.pdf]

Table S5

Query name	Predicted functional class (FC)	FC prediction probability	Predicted substrate specificity (SS)	SS prediction probability
**C1 AMP_binding**	Aryl-CoA ligase	**0.48**	cinnamate and succinylbenzoate derivatives	**0.34**
**C2 AMP_binding**	Long chain acyl-CoA synthetase	**0.54**	C13 through C17	**0.45**
**BGC0000871_MXAN_1528_AMP-binding**	Long chain acyl-CoA synthetase	**0.67**	C13 through C17	**0.6**
