# Supplementary material for: Comparative Metagenomic Analysis of Biosynthetic Diversity across Sponge Microbiomes Highlights Metabolic Novelty, Conservation, and Diversification
Source: mSystems. 2022 Jul 18;7(4):e00357-22. doi: 10.1128/msystems.00357-22 (PMC9426513; doi:10.1128/msystems.00357-22)
Supplement: TABLE S1 [file msystems.00357-22-s0004.pdf]

Table S1

Name	Type	Species	Depth_avg (m)	Location	Coordinates	Country	Sampling Year	Sequencing Year	Sequencing technology
Aply16	Sponge Tissue	Aplysina aerophoba	10,25	Cala Montgo	N42°06'52.20", E3°10'06.52"	Spain	2014	2014	Hybrid Illumina Pacbio
Aply21	Sponge Tissue	Aplysina aerophoba	10,25	Cala Montgo	N42°06'52.20", E3°10'06.52"	Spain	2014	2014	Hybrid Illumina Pacbio
Aply22	Sponge Tissue	Aplysina aerophoba	10,25	Cala Montgo	N42°06'52.20", E3°10'06.52"	Spain	2014	2014	Hybrid Illumina Pacbio
Aply23	Sponge Tissue	Aplysina aerophoba	10,25	Cala Montgo	N42°06'52.20", E3°10'06.52"	Spain	2014	2014	Hybrid Illumina Pacbio
Pf4	Sponge Tissue	Petrocia ficiformis	5,5	Sfakia, Crete (Semi-dark zone)	N35°12'0.7", E24°7'09.8"	Greece	2018	2018	Illumina
Pf5	Sponge Tissue	Petrocia ficiformis	5,5	Sfakia, Crete (Semi-dark zone)	N35°12'0.7", E24°7'09.8"	Greece	2018	2018	Illumina
Pf6	Sponge Tissue	Petrocia ficiformis	5,5	Sfakia, Crete (Semi-dark zone)	N35°12'0.7", E24°7'09.8"	Greece	2018	2018	Illumina
Pf7	Sponge Tissue	Petrocia ficiformis	5,5	Sfakia, Crete (Dark zone)	N35°12'0.7", E24°7'09.8"	Greece	2018	2018	Illumina
Pf8	Sponge Tissue	Petrocia ficiformis	5,5	Sfakia, Crete (Dark zone)	N35°12'0.7", E24°7'09.8"	Greece	2018	2018	Illumina
Pf9	Sponge Tissue	Petrocia ficiformis	5,5	Sfakia, Crete (Dark zone)	N35°12'0.7", E24°7'09.8"	Greece	2018	2018	Illumina
Pf10	Sponge Tissue	Petrocia ficiformis	5,5	Sfakia, Crete (Entrance)	N35°12'0.7", E24°7'09.8"	Greece	2018	2018	Illumina
Pf11	Sponge Tissue	Petrocia ficiformis	5,5	Sfakia, Crete (Entrance)	N35°12'0.7", E24°7'09.8"	Greece	2018	2018	Illumina
Pf12	Sponge Tissue	Petrocia ficiformis	5,5	Sfakia, Crete (Entrance)	N35°12'0.7", E24°7'09.8"	Greece	2018	2018	Illumina
gb1	Sponge Tissue	Geodia barretti	450	Scengsbukt-Korsfjord	N60°8'8", E5°6'42"	Norway	2017	2018	Illumina
gb2_2	Sponge Tissue	Geodia barretti	150	Scengsbukt-Korsfjord	N60°8'8", E5°6'42"	Norway	2017	2018	Illumina
gb4_2	Sponge Tissue	Geodia barretti	150	Scengsbukt-Korsfjord	N60°8'8", E5°6'42"	Norway	2017	2018	Illumina
gb5_2	Sponge Tissue	Geodia barretti	150	Scengsbukt-Korsfjord	N60°8'8", E5°6'42"	Norway	2017	2018	Illumina
gb6	Sponge Tissue	Geodia barretti	150	Scengsbukt-Korsfjord	N60°8'8", E5°6'42"	Norway	2017	2018	Illumina
gb7	Sponge Tissue	Geodia barretti	450	Scengsbukt-Korsfjord	N60°8'8", E5°6'42"	Norway	2017	2018	Illumina
gb8_2	Sponge Tissue	Geodia barretti	450	Scengsbukt-Korsfjord	N60°8'8", E5°6'42"	Norway	2017	2018	Illumina
gb9	Sponge Tissue	Geodia barretti	450	Scengsbukt-Korsfjord	N60°8'8", E5°6'42"	Norway	2017	2018	Illumina
gb10	Sponge Tissue	Geodia barretti	450	Scengsbukt-Korsfjord	N60°8'8", E5°6'42"	Norway	2017	2018	Illumina
gb126	Sponge Tissue	Geodia barretti	1213	Davis Strait	N62°52'15.1", W58°37'34.32"	Canada	2015	2018	Illumina
gb278	Sponge Tissue	Geodia barretti	1335	Davis Strait	N61°53'36.13", W60°7'57.612"	Canada	2014	2018	Illumina
gb305	Sponge Tissue	Geodia barretti	1437	Davis Strait	N62°31'6.24", W59°58'13.872"	Canada	2014	2018	Illumina
gb1_f	Filtered Sea Water	N.a. Seawater Atl	150	Scengsbukt-Korsfjord	N60°8'8", E5°6'42"	Norway	2017	2018	Illumina
gb2_f	Filtered Sea Water	N.a. Seawater Atl	150	Scengsbukt-Korsfjord	N60°8'8", E5°6'42"	Norway	2017	2018	Illumina
gb3_f	Filtered Sea Water	N.a. Seawater Atl	150	Scengsbukt-Korsfjord	N60°8'8", E5°6'42"	Norway	2017	2018	Illumina
gb5_6_f	Filtered Sea Water	N.a. Seawater Atl	450	Scengsbukt-Korsfjord	N60°8'8", E5°6'42"	Norway	2017	2018	Illumina
gb9_f	Filtered Sea Water	N.a. Seawater Atl	450	Scengsbukt-Korsfjord	N60°8'8", E5°6'42"	Norway	2017	2018	Illumina
gb10_f	Filtered Sea Water	N.a. Seawater Atl	450	Scengsbukt-Korsfjord	N60°8'8", E5°6'42"	Norway	2017	2018	Illumina
sw_7	Filtered Sea Water	N.a. Seawater Med	10,25	Cala Montgo	N42°06'52.20", E3°10'06.52"	Spain	2014	2014	Illumina
sw_8	Filtered Sea Water	N.a. Seawater Med	10,25	Cala Montgo	N42°06'52.20", E3°10'06.52"	Spain	2014	2014	Illumina
sw_9	Filtered Sea Water	N.a. Seawater Med	10,25	Cala Montgo	N42°06'52.20", E3°10'06.52"	Spain	2014	2014	Illumina
