# Supplementary material for: Comparative Metagenomic Analysis of Biosynthetic Diversity across Sponge Microbiomes Highlights Metabolic Novelty, Conservation, and Diversification
Source: mSystems. 2022 Jul 18;7(4):e00357-22. doi: 10.1128/msystems.00357-22 (PMC9426513; doi:10.1128/msystems.00357-22)
Supplement: TABLE S3 [file msystems.00357-22-s0006.pdf]

Table S3

Genome	Completeness	Contamination	Genome	Completeness	Contamination
**gb2_2_bin.60.fa**	85.73	0.8	**Pf11_bin.25.fa**	86.8	0.99
**Pf4_bin.57.fa**	82.8	4.53	**Aply16_bin.9.fa**	95.44	0.99
**Pf7_bin.11.fa**	96.0	4.58	**gb278_bin.73.fa**	91.55	0.0
**Pf5_bin.33.fa**	79.62	0.83	**gb278_bin.64.fa**	88.67	0.0
**Pf12_bin.26.fa**	87.6	5.25	**gb4_2_bin.32.fa**	93.95	0.0
**gb5_6_f_bin.4.fa**	94.29	1.18	**Pf8_bin.20.fa**	96.59	0.0
**gb10_f_bin.41.fa**	90.26	1.71	**Aply16_bin.3.fa**	96.59	0.0
**gb_f_9_bin.4.fa**	90.26	0.0	**Pf9_bin.30.fa**	94.61	0.0
**gb5_6_f_bin.16.fa**	93.49	1.08	**Pf10_bin.13.fa**	81.65	0.0
**gb10_f_bin.38.fa**	94.76	4.82	**Aply22_bin.4.fa**	86.69	0.0
**Pf7_bin.57.fa**	92.57	0.04	**Pf4_bin.24.fa**	95.73	1.71
**Pf9_bin.46.fa**	93.73	0.68	**gb1_bin.31.fa**	90.26	2.56
**Pf5_bin.30.fa**	97.07	0.34	**gb126_bin.75.fa**	89.46	1.71
**gb5_6_f_bin.55.fa**	87.01	2.24	**gb8_2_bin.30.fa**	93.07	0.0
**Pf4_bin.22.fa**	89.08	0.0	**gb126_bin.35.fa**	77.59	0.0
**Pf5_bin.48.fa**	93.89	1.1	**gb5_2_bin.68.fa**	92.02	0.0
**gb305_bin.41.fa**	93.34	3.85	**Pf10_bin.11.fa**	98.29	2.56
**Pf10_bin.43.fa**	95.54	3.3	**gb278_bin.39.fa**	90.12	2.28
**gb5_6_f_bin.58.fa**	95.54	3.3	**gb126_bin.36.fa**	76.84	0.43
**gb10_f_bin.37.fa**	98.47	2.2	**gb305_bin.7.fa**	90.46	7.26
**Pf9_bin.12.fa**	92.12	1.1	**gb7_bin.42.fa**	94.98	3.42
**gb7_bin.51.fa**	95.54	2.2	**gb7_bin.28.fa**	83.52	0.9
**Pf8_bin.23.fa**	100.0	3.36	**Pf8_bin.46.fa**	90.81	0.85
**Pf9_bin.53.fa**	90.11	2.75	**gb126_bin.28.fa**	80.79	6.03
**gb4_2_bin.61.fa**	77.59	0.0	**Pf5_bin.39.fa**	89.79	1.88
**Pf11_bin.3.fa**	83.82	2.2	**gb6_bin.52.fa**	84.62	0.85
**gb305_bin.78.fa**	92.86	3.3	**Pf4_bin.17.fa**	91.83	0.85
**Aply22_bin.19.fa**	75.34	3.45	**gb5_2_bin.9.fa**	93.59	2.56
**Pf10_bin.2.fa**	97.8	3.3	**Pf11_bin.39.fa**	93.16	1.03
**gb8_2_bin.8.fa**	89.41	4.46	**gb7_bin.52.fa**	81.39	1.07
**gb305_bin.35.fa**	95.6	3.3	**gb305_bin.22.fa**	75.86	0.0
**gb3_2_bin.69.fa**	96.7	0.0	**gb126_bin.47.fa**	80.89	1.76
**Pf5_bin.5.fa**	97.74	1.1	**gb305_bin.51.fa**	89.9	3.42
**Pf9_bin.15.fa**	97.74	0.0	**Pf5_bin.19.fa**	82.76	0.0
**Pf12_bin.20.fa**	96.64	0.55	**Pf7_bin.19.fa**	87.98	1.71
**gb305_bin.13.fa**	95.6	0.3	**Aply16_bin.4.fa**	79.33	1.93

**Pf7_bin.67.fa**	97.8	0.3	**gb126_bin.26.fa**	92.9	2.74
**gb10_bin.62.fa**	93.34	1.1	**gb126_bin.18.fa**	78.32	1.71
**Pf7_bin.5.fa**	91.14	0.0	**Pf5_bin.57.fa**	95.85	1.82
**gb3_2_bin.44.fa**	82.13	2.26	**gb8_2_bin.43.fa**	86.0	0.91
**Pf7_bin.59.fa**	96.09	1.4	**Aply22_bin.37.fa**	95.85	2.73
**gb1_bin.4.fa**	86.16	2.2	**gb5_2_bin.13.fa**	87.98	0.85
**Aply21_bin.5.fa**	81.59	2.5	**gb305_bin.19.fa**	94.34	1.28
**gb_2_f_bin.4.fa**	83.52	0.0	**Pf4_bin.21.fa**	93.16	3.42
**Pf12_bin.35.fa**	96.58	1.64	**gb5_2_bin.32.fa**	95.73	1.71
**Pf7_bin.14.fa**	93.5	0.56	**gb278_bin.63.fa**	93.16	2.56
**Aply22_bin.9.fa**	94.92	1.13	**Pf12_bin.44.fa**	82.82	1.73
**Pf7_bin.31.fa**	93.48	1.98	**Pf11_bin.13.fa**	96.59	1.68
**gb278_bin.3.fa**	94.33	1.13	**Aply16_bin.23.fa**	95.75	1.68
**Pf4_bin.44.fa**	92.92	1.13	**gb1_bin.7.fa**	93.22	0.84
**Pf12_bin.28.fa**	98.39	0.54	**gb5_2_bin.46.fa**	80.25	0.0
**sw_9_bin.4.fa**	75.33	0.54	**gb5_2_bin.36.fa**	91.55	2.52
**sw_9_bin.11.fa**	92.03	3.76	**gb278_bin.37.fa**	92.51	1.68
**Aply22_bin.13.fa**	97.06	0.0	**gb3_2_bin.16.fa**	91.34	3.36
**sw_9_bin.14.fa**	84.93	1.75	**Pf11_bin.31.fa**	93.23	3.36
**sw_8_bin.5.fa**	95.06	1.53	**gb5_6_f_bin.30.fa**	86.68	1.31
**gb5_6_f_bin.25.fa**	93.55	2.66	**gb10_f_bin.32.fa**	75.69	0.89
**Pf7_bin.52.fa**	91.83	1.33	**gb_1_f_bin.35.fa**	88.19	2.37
**gb4_2_bin.25.fa**	84.45	5.49	**Pf9_bin.23.fa**	84.67	0.0
**gb3_2_bin.45.fa**	80.16	3.3	**Pf12_bin.13.fa**	90.18	0.0
**Pf8_bin.6.fa**	96.64	3.6	**Pf6_bin.41.fa**	88.34	0.0
**Pf5_bin.13.fa**	95.48	4.7	**Pf5_bin.15.fa**	80.06	1.79
**Pf5_bin.31.fa**	94.34	2.2	**Aply22_bin.15.fa**	90.34	0.43
**gb4_2_bin.48.fa**	79.06	0.0	**sw_7_bin.3.fa**	94.82	0.58
**Aply22_bin.11.fa**	97.58	2.25	**gb_f_9_bin.8.fa**	99.53	0.94
**gb4_2_bin.50.fa**	86.22	1.23	**sw_7_bin.7.fa**	96.08	0.06
**Pf8_bin.49.fa**	87.68	6.74	**gb_f_9_bin.7.fa**	100.0	0.0
**Pf10_bin.3.fa**	94.24	4.4	**Aply23_bin.14.fa**	84.44	0.43
**Pf12_bin.33.fa**	97.48	4.9	**Pf4_bin.61.fa**	92.38	0.8
**gb5_2_bin.34.fa**	77.82	2.25	**gb305_bin.70.fa**	95.71	1.28
**gb2_2_bin.46.fa**	76.66	1.1	**gb5_6_f_bin.54.fa**	81.26	3.44
**Pf9_bin.24.fa**	80.73	2.25	**Aply22_bin.39.fa**	87.21	1.88
**Pf5_bin.52.fa**	93.09	4.49	**sw_9_bin.8.fa**	98.17	1.24
**Aply23_bin.19.fa**	96.46	3.37	**sw_9_bin.6.fa**	96.58	0.44

**Pf9_bin.10.fa**	93.14	4.4	**sw_7_bin.14.fa**	84.38	2.18
**Pf5_bin.37.fa**	82.75	0.0	**sw_7_bin.8.fa**	87.83	1.64
**Pf5_bin.4.fa**	95.38	5.49	**Aply16_bin.13.fa**	82.87	1.51
**Aply16_bin.2.fa**	96.36	3.37	**Pf9_bin.57.fa**	99.0	0.0
**gb7_bin.5.fa**	84.35	2.2	**Aply22_bin.45.fa**	92.74	0.5
**Pf9_bin.14.fa**	97.42	4.49	**gb278_bin.18.fa**	93.64	0.5
**Pf11_bin.65.fa**	95.34	4.4	**Pf7_bin.61.fa**	92.45	0.79
**Pf4_bin.36.fa**	97.74	0.0	**gb2_2_bin.49.fa**	88.09	0.03
**Aply23_bin.13.fa**	97.15	0.0	**Aply22_bin.44.fa**	75.74	2.17
**gb126_bin.52.fa**	83.66	0.85	**gb5_6_f_bin.61.fa**	88.59	1.27
**Pf11_bin.32.fa**	90.02	1.28	**gb305_bin.21.fa**	96.23	0.43
**gb10_f_bin.14.fa**	78.82	1.3	**gb8_2_bin.68.fa**	89.37	0.52
**gb10_f_bin.44.fa**	99.15	2.23	**Pf11_bin.50.fa**	96.96	0.43
**gb305_bin.8.fa**	86.32	2.31	**gb8_2_bin.1.fa**	95.02	0.17
**Pf7_bin.64.fa**	98.29	2.14	**Pf4_bin.37.fa**	94.43	0.5
**Aply22_bin.36.fa**	92.31	2.14	**sw_7_bin.10.fa**	99.0	0.03
**Aply22_bin.5.fa**	83.05	2.99	**Aply16_bin.21.fa**	95.38	0.0
**gb8_2_bin.58.fa**	90.6	1.28	**gb278_bin.34.fa**	94.54	0.0
**Pf9_bin.45.fa**	94.44	1.28	**Pf9_bin.40.fa**	94.42	0.84
**Pf6_bin.60.fa**	96.58	1.28	**Pf10_bin.9.fa**	96.1	0.0
**Aply16_bin.8.fa**	80.12	3.5	**gb5_2_bin.33.fa**	94.42	1.22
**Aply23_bin.11.fa**	88.89	2.35	**gb_f_9_bin.17.fa**	82.63	2.17
**Pf9_bin.13.fa**	92.74	2.14	**gb5_6_f_bin.31.fa**	80.99	1.22
**Pf10_bin.8.fa**	95.73	1.28	**gb_f_3_bin.30.fa**	89.02	0.61
**gb5_6_f_bin.18.fa**	81.03	1.28	**Pf4_bin.35.fa**	77.69	1.09
**gb_f_9_bin.31.fa**	97.34	0.85	**gb305_bin.15.fa**	80.29	3.43
**gb10_f_bin.45.fa**	90.5	2.14	**gb7_bin.36.fa**	75.56	1.61
**Aply23_bin.8.fa**	96.15	1.28	**gb305_bin.79.fa**	87.2	0.41
**gb5_2_bin.28.fa**	92.99	1.28	**Pf8_bin.36.fa**	78.45	3.3
**Aply22_bin.47.fa**	88.89	1.28	**gb278_bin.45.fa**	85.61	3.76
**gb5_2_bin.63.fa**	93.16	1.28	**Pf8_bin.5.fa**	86.0	5.11
**gb5_2_bin.60.fa**	81.48	1.28	**gb_1_f_bin.10.fa**	97.51	0.59
**Pf7_bin.8.fa**	89.32	0.43	**Pf9_bin.9.fa**	84.94	7.84
**Aply22_bin.32.fa**	94.87	1.57	**gb9_bin.59.fa**	79.71	1.99
**Pf10_bin.10.fa**	92.12	0.0	**Pf5_bin.16.fa**	90.23	1.72
**gb126_bin.71.fa**	83.64	1.16	**gb_f_9_bin.12.fa**	79.38	0.0
**gb126_bin.20.fa**	81.36	0.91	**gb_2_f_bin.27.fa**	78.37	0.33
**Aply22_bin.17.fa**	97.27	1.82	**gb_f_9_bin.10.fa**	82.9	0.45

**gb5_6_f_bin.40.fa**	85.09	1.19	**gb3_2_bin.25.fa**	83.26	0.0
**gb126_bin.3.fa**	77.73	0.0	**Pf5_bin.27.fa**	77.92	0.56
**gb126_bin.13.fa**	84.92	2.1	**Pf9_bin.7.fa**	85.8	1.48
**Aply22_bin.41.fa**	90.91	0.91	**Aply22_bin.18.fa**	84.26	1.11
**gb305_bin.38.fa**	91.82	0.93	**gb126_bin.66.fa**	84.13	2.21
**gb6_bin.59.fa**	87.65	0.21	**Pf7_bin.62.fa**	82.87	1.71
**gb305_bin.52.fa**	82.01	0.0	**Pf6_bin.10.fa**	81.8	0.91
**gb305_bin.54.fa**	85.03	0.46	**Pf11_bin.40.fa**	83.4	1.33
**Pf4_bin.62.fa**	83.18	0.0	**Pf6_bin.2.fa**	79.59	0.74
**gb10_f_bin.34.fa**	75.02	1.43	**Pf7_bin.23.fa**	85.49	1.54
**Pf5_bin.43.fa**	94.39	0.99	**gb3_2_bin.14.fa**	75.37	2.72
**Pf12_bin.2.fa**	91.09	0.0	**gb278_bin.58.fa**	85.73	0.31
**gb10_f_bin.50.fa**	90.76	0.0	**gb_1_f_bin.30.fa**	92.21	0.77
**gb10_f_bin.10.fa**	78.62	6.16	**sw_8_bin.2.fa**	100.0	0.84
**Pf6_bin.31.fa**	91.75	0.99	**sw_8_bin.15.fa**	98.42	0.67
**gb5_2_bin.49.fa**	89.77	0.99	**gb_f_3_bin.27.fa**	93.06	0.21
**Aply22_bin.46.fa**	90.96	0.99	**sw_8_bin.14.fa**	85.81	0.5
**gb278_bin.5.fa**	86.8	2.64	**gb5_6_f_bin.7.fa**	84.53	2.33
**gb3_2_bin.67.fa**	83.99	0.0	**gb278_bin.6.fa**	81.68	1.63
**Pf6_bin.23.fa**	93.23	0.0	**gb_f_3_bin.12.fa**	89.69	0.49
**gb10_f_bin.46.fa**	77.05	1.98	**Pf4_bin.53.fa**	91.42	1.11
**gb5_2_bin.22.fa**	78.38	1.21	**Pf10_bin.4.fa**	92.16	0.0
**Pf5_bin.47.fa**	91.09	0.09	**gb10_f_bin.30.fa**	79.99	1.73
**Aply22_bin.34.fa**	94.22	0.99	**gb126_bin.27.fa**	82.17	0.88
**gb_f_9_bin.20.fa**	80.64	0.0	**sw_9_bin.16.fa**	77.88	3.59
**gb5_6_f_bin.38.fa**	78.07	3.19	**sw_9_bin.13.fa**	93.17	0.0
**gb5_6_f_bin.1.fa**	94.45	1.39	**gb278_bin.54.fa**	83.43	0.2
**Pf7_bin.1.fa**	83.83	0.0	**gb278_bin.19.fa**	95.4	1.98
**Pf4_bin.27.fa**	89.77	0.99	**gb126_bin.31.fa**	78.67	1.98
**gb7_bin.40.fa**	78.79	0.0	**gb5_2_bin.7.fa**	87.29	0.99
**gb5_2_bin.5.fa**	90.76	2.97	**Pf10_bin.55.fa**	84.82	1.98
**gb126_bin.73.fa**	89.71	1.32	**gb1_bin.53.fa**	93.73	0.99
**Pf9_bin.39.fa**	85.96	0.99	**Pf9_bin.38.fa**	81.95	1.98
**Aply23_bin.1.fa**	88.78	2.64	**gb7_bin.12.fa**	88.78	3.16
**Pf9_bin.5.fa**	95.51	0.99	**gb7_bin.46.fa**	92.24	1.98
**Aply22_bin.12.fa**	97.2	3.41	**gb305_bin.80.fa**	94.72	0.44
**Aply21_bin.3.fa**	95.51	0.99	**Pf10_bin.14.fa**	75.17	0.5
**Pf8_bin.4.fa**	96.7	0.0	**Pf5_bin.25.fa**	95.71	0.0

**Aply22_bin.16.fa**	90.76	1.98	**gb8_2_bin.5.fa**	90.17	0.0
**gb278_bin.49.fa**	80.27	0.0	**gb5_2_bin.42.fa**	91.96	0.0
**gb278_bin.16.fa**	82.64	9.11	**gb3_2_bin.9.fa**	97.69	0.0
**gb126_bin.45.fa**	75.74	8.03	**gb126_bin.48.fa**	89.77	0.99
**gb7_bin.2.fa**	79.67	0.0	**gb278_bin.59.fa**	81.63	0.0
**gb305_bin.37.fa**	87.1	0.0	**gb1_bin.54.fa**	90.76	0.0
**Pf7_bin.54.fa**	89.77	0.5	**gb5_2_bin.54.fa**	86.8	0.0
**gb126_bin.67.fa**	92.5	0.0	**Pf7_bin.34.fa**	82.18	1.98
