# Supplementary material for: Comparative Metagenomic Analysis of Biosynthetic Diversity across Sponge Microbiomes Highlights Metabolic Novelty, Conservation, and Diversification
Source: mSystems. 2022 Jul 18;7(4):e00357-22. doi: 10.1128/msystems.00357-22 (PMC9426513; doi:10.1128/msystems.00357-22)
Supplement: TABLE S6 [file msystems.00357-22-s0009.pdf]

Table S6

Taxa	GCF/MAG	5496	380	3574	6120	4988	3047	4093	172	4034
Acidobacteria	Pf5_bin.39	x								
	Pf4_bin.17	x	x							
	gb6_bin.52	x	x							
	gb126_bin.28	x								
Poribacteria	Pf8_bin.6			x						
	Pf5_bin.13			x						
	Aply16_bin.2				x					
	Pf10_bin.3				x	x				
	Pf11_bin.65				x					
	gb4_2_bin.50				x	x				
	Pf12_bin.33					x	x			
	Pf5_bin.4					x				
	Pf8_bin.49					x				
	gb5_2_bin.34					x				
	Pf5_bin.37						x			
	Pf9_bin.14						x			
	Pf9_bin.10						x			
	Aply23_bin.19							x		
	Pf5_bin.52							x		
	Pf9_bin.24							x		
	Aply22_bin.11								x	
	Pf5_bin.31									x
